# Genetic polymorphisms in genes regulating cell death and prognosis of patients with rectal cancer receiving postoperative chemoradiotherapy

**DOI:** 10.20892/j.issn.2095-3941.2022.0711

**Published:** 2023-05-04

**Authors:** Hongxia Chen, Luxi Yin, Jie Yang, Ningxin Ren, Jinna Chen, Qixuan Lu, Ying Huang, Yanru Feng, Weihu Wang, Shulian Wang, Yueping Liu, Yongwen Song, Yexiong Li, Jing Jin, Wen Tan, Dongxin Lin

**Affiliations:** 1 State Key Laboratory of Molecular Oncology, Department of Etiology and Carcinogenesis, Beijing Key Laboratory for Carcinogenesis and Cancer Prevention, National Cancer Center/National Clinical Research Center for Cancer/Cancer Hospital, Chinese Academy of Medical Sciences and Peking Union Medical College, Beijing 100021, China; 2 Department of Radiation Oncology, National Cancer Center/National Clinical Research Center for Cancer/Cancer Hospital, Chinese Academy of Medical Sciences and Peking Union Medical College, Beijing 100021, China; 3 Sun Yat-sen University Cancer Center, State Key Laboratory of Oncology in South China, Guangzhou 510060, China

**Keywords:** Rectal neoplasms, genetic variation, regulated cell death, overall survival, ALOX5

## Abstract

**Objective:**

The identification of biomarkers for predicting chemoradiotherapy efficacy is essential to optimize personalized treatment. This study determined the effects of genetic variations in genes involved in apoptosis, pyroptosis, and ferroptosis on the prognosis of patients with locally advanced rectal cancer receiving postoperative chemoradiotherapy (CRT).

**Methods:**

The Sequenom MassARRAY was used to detect 217 genetic variations in 40 genes from 300 patients with rectal cancer who received postoperative CRT. The associations between genetic variations and overall survival (OS) were evaluated using hazard ratios (HRs) and 95% confidence intervals (CIs) computed using a Cox proportional regression model. Functional experiments were performed to determine the functions of the arachidonate 5-lipoxygenase (*ALOX5*) gene and the *ALOX5* rs702365 variant.

**Results:**

We detected 16 genetic polymorphisms in *CASP3*, *CASP7*, *TRAILR2*, *GSDME*, *CASP4*, *HO-1*, *ALOX5*, *GPX4*, and *NRF2* that were significantly associated with OS in the additive model (*P* < 0.05). There was a substantial cumulative effect of three genetic polymorphisms (*CASP4* rs571407, *ALOX5* rs2242332, and *HO-1* rs17883419) on OS. Genetic variations in the *CASP4* and *ALOX5* gene haplotypes were associated with a higher OS. We demonstrated, for the first time, that rs702365 [G] > [C] represses *ALOX5* transcription and corollary experiments suggested that *ALOX5* may promote colon cancer cell growth by mediating an inflammatory response.

**Conclusions:**

Polymorphisms in genes regulating cell death may play essential roles in the prognosis of patients with rectal cancer who are treated with postoperative CRT and may serve as potential genetic biomarkers for individualized treatment.

## Introduction

Colorectal cancer (CRC), a major malignant disease of the gastrointestinal tract, has the third highest incidence among cancers and is the second leading cause of cancer deaths^1^. The incidence of CRC ranks fourth among men and third among women in China^2^. CRC is caused by interactions between genetic and environmental factors^3^. Pathogenic and putative pathogenic germline mutations can increase the risk of CRC^4^. Rectal cancer accounts for approximately 30% of CRC cases; however, the treatment for resectable stage II and III rectal cancer is different from colon cancer, largely because of the different local recurrence patterns^5,6^. Postoperative chemoradiotherapy (CRT) is often administered to patients with locally advanced rectal cancer; however, the efficacy varies from one individual to another^7^. Therefore, the identification of prognostic markers is important for personalized treatments.

Cell death can be categorized into accidental death (ACD) and regulated death (RCD)^8^. ACD is a biological process in which cells die uncontrollably, whereas RCD is regulated by a set of molecules. Under physiologic conditions, RCD is also known as programmed cell death (PCD). RCD includes apoptosis, pyroptosis, and ferroptosis, which have been widely studied and exhibit unique molecular mechanisms^9^. Apoptosis is a well-recognized form of PCD that is essential for the normal development and function of organisms; however, aberrant apoptosis is associated with many diseases, including cancer and autoimmune diseases^10^. Radiotherapy and chemotherapy induce tumor cell death mainly through caspase-dependent apoptosis^11,12^; however, radiotherapy and chemotherapy also induce pyroptosis and ferroptosis to exert anti-tumor effects^13–18^. Pyroptosis is an inflammatory RCD in which plasma membrane pores are formed by members of the gasdemin protein family that are often cleaved by activated caspases, such as caspase-1 (CASP1), caspase-4 (CASP4), caspase-5 (CASP5), and caspase-11 (CASP11)^9,19^. Ferroptosis is a form of RCD caused by phospholipid peroxidation, which is dependent on iron, reactive oxygen species (ROS), and phospholipids containing polyunsaturated fatty acid chains (PUFA-PL)^20,21^. Ferroptosis is regulated by the antioxidant enzyme glutathione peroxidase 4 (GPX4), iron chelators, and antioxidants^22,23^. Induction of ferroptosis holds great promise in tumor therapy because the inducers of ferroptosis were discovered in the process of searching for new cancer therapeutic compounds^24,25^.

Single-nucleotide polymorphisms (SNPs) are associated with the prognosis of patients with cancer. We have previously shown that genetic variations, such as SNPs in microRNA and DNA repair genes, are significantly associated with the prognosis of patients with locally advanced rectal cancer who are treated with postoperative CRT^26,27^. Moreover, polymorphisms in genes involved in the apoptosis and ferroptosis pathways are associated with the overall survival (OS) of patients with cancer^28–30^; however, previous studies only focused on one or two genes. A comprehensive understanding of the association between OS and ferroptosis and apoptosis pathways is limited.

In the present study we investigated the associations between haplotype-tagging SNPs (htSNPs) of key genes involved in the apoptosis, pyroptosis, and ferroptosis pathways with the OS of rectal cancer patients treated with postoperative CRT. We analyzed the associations with single locus, combined multiple loci, and haplotypes in these three pathways. What's more, this is the first study to demonstrate that arachidonate lipoxygenase 5 (*ALOX5*) rs702365 [G] > [C] represses *ALOX5* transcription and leads to a decrease in ALOX5 expression.

## Materials and methods

### Patient characteristics and data collection

Three hundred patients with rectal cancer receiving concurrent CRT were enrolled in this study. The patient characteristics have been described in our previous studies^26,27^. All patients signed an informed consent form. This study was approved by the Institutional Review Board of the Cancer Hospital (Chinese Academy of Medical Sciences; IRB No. NCC2019C-145) and met the Declaration of Helsinki requirements. Briefly, the criteria for enrollment were as follows: (1) diagnosis of rectal adenocarcinoma by pathology experts at the Cancer Hospital of the Chinese Academy of Medical Sciences from January 2005 to June 2015; (2) primary and locally advanced rectal cancer without distant metastasis; (3) Karnofsky performance score (KPS) ≥ 70 and life expectancy ≥ 6 months; (4) age ≤ 75 years; (5) normal routine blood and biochemistry tests before concurrent CRT; (6) no history of CRT or other tumors; and (7) the patient underwent total mesorectal excision surgery and concurrent CRT. The total radiation dose was as follows: 50 Gy; 2 Gy/session; and 5 sessions/week for 5 weeks. The chemotherapeutic regimen was capecitabine (1,600 mg/m^2^ daily administered orally twice for 2 weeks, stopped for 1 week, and continued for 2 weeks).

The relevant clinical data of the patients were obtained from medical records, hematologic tests, imaging results, and telephone inquiries about cancer-related health status after discharge from the hospital. The last follow-up date was April 1, 2021.

### Screening for genetic variations

We adopted the candidate gene strategy to select genes. First, we chose genes that are thought to be involved in the apoptosis, pyroptosis, and ferroptosis pathways based on a literature search in PubMed. Second, we selected the genes that have been reported to be related to radiotherapy or chemotherapy sensitivity, or have been more studied from the genes identified in the first step. After the two steps, we finally selected 40 candidate genes, including 17 genes involved in the apoptosis pathway (*APAF1, BAK, BAX, BCL2, BID, CASP3, CASP6, CASP7, CASP8, CASP9, CASP10, FAS, FASL, TNFR, TRAIL, TRAILR1*, and *TRAILR2*), 8 genes involved in the pyroptosis pathway (*AIM2, CASP1, CASP4, CASP5, CASP11, GSDMD, GSDME*, and *NLRP3*), and 15 genes involved in the ferroptosis pathway (*ALOX15, ALOX5, BECN1, DMT1, EIF2S1, FADS2, FTH1, GPX4, HO-1, NQO1, NRF2, PTGS2, SLC3A2, SLC7A11*, and *TFRC*).

The genetic variation screening strategy is shown in **Supplementary Figure S1**. Among the loci of 40 candidate genes, SNPs with a minor allele frequency (MAF) ≥ 0.1 in the Chinese Han Beijing population (CHB) of the Thousand Genomes Project (1,000 Genomes) query were first selected. For SNPs with a correlation coefficient (*r*^2^) ≥ 0.8, only 1 SNP was selected as the htSNP based on linkage disequilibrium (LD) information of the CHB in the database, and 217 genetic variations were selected for genotyping. Finally, we analyzed the associations of 169 SNPs with the following criteria: Hardy–Weinberg equilibrium (HWE) > 0.05; MAF ≥ 0.10; and call rate ≥ 95%.

### Genotyping

Genomic DNA was extracted from blood samples (2 mL) collected from each patient before CRT. Genotyping was performed using the Sequenom MassARRAY method, with one blank sample and four duplicate samples arranged in a 96-well plate for quality control.

### Cell lines and reagents

HCT8 and HCT116 cells were purchased from the Cell Bank of the Institute of Basic Medical Sciences (Chinese Academy of Medical Sciences) and the School of Basic Medicine (Peking Union Medical College). These cell lines were tested for *Mycoplasma* contamination and authenticated using STR profiling. HCT8 cells were maintained in RPMI-1640 medium, whereas HCT116 cells were maintained in DMEM with 10% fetal bovine serum in a 5% CO_2_ humidified atmosphere at 37°C.

### Construction of plasmids, transient transfection, and dual-luciferase reporter assays

A total of 540-bp DNA fragments surrounding the functional candidate SNP rs702365 [G] or [C] alleles were cloned into the pGL4.10-SV40 firefly luciferase expression vector. To determine whether the DNA fragment containing the rs702365 [G] or [C] allele had different *ALOX5* promoter-driving abilities, the SV40 sequence in the plasmid was replaced with the *ALOX5* promoter (**Supplementary Table S1**). The authenticity of all constructs was verified by DNA sequencing. The primers used for plasmid construction are listed in **Supplementary Table S1**. For dual-luciferase reporter gene assays, 8 × 10^4^ HCT8 cells or 1 × 10^5^ HCT116 cells were seeded in 48-well plates and transfected with 300 ng of allele-different reporter constructs per well using Lipofectamine 2000 (Invitrogen, Thermo Fisher Scientific, Waltham, MA, USA) after 16 h. The Renilla luciferase plasmid, pRL-TK (6 ng; Promega, Madison, WI, USA), was co-transfected in each well as an internal control. After a 24 h transfection, the cells were collected and analyzed for luciferase activity. Each plasmid construct had three replicates and was used in at least three independent experiments. The pGL4.10-SV40 vector and pRL-TK plasmid maps are shown in **Supplementary Figure S2A, S2B**.

### Electrophoretic mobility-shift assays (EMSAs)

Nuclear proteins were extracted from HCT8 and HCT116 cells using a Nuclear Protein Extraction Kit (Thermo Fisher Scientific). Additionally, 25-bp double-stranded oligonucleotides containing rs702365[G] or rs702365[C] were synthesized and labeled with biotin at the 5′ end (**Supplementary Table S1**). Nuclear extracts (10 μg of protein) were incubated with 100 fmol of biotin-labeled oligonucleotide probes for 20 min using the Chemiluminescent EMSA kit (Beyotime Biotechnology, Shanghai, China). Unlabeled oligonucleotides were added before adding biotin-labeled probes for competition assays. After electrophoreses at 110 V in 0.5 × TBE for 95 min, samples were transferred onto a nylon membrane (Millipore Sigma, Burlington, VT, USA) in 0.5 × TBE at 380 mA for 40 min. The transferred DNA was cross-linked to the membrane at 120 mJ/cm^2^ for 90 s, then detected using the ECL reagent in the EMSA kit.

### Small-interfering RNA transduction and quantitative real-time polymerase chain reaction (RT-qPCR) assays

HCT8 (3 × 10^5^) or HCT116 (6 × 10^5^) cells were seeded in 6-well plates and transfected with small-interfering RNAs (siRNAs) targeting *ALOX5* or a negative control (siControl) siRNA using Lipofectamine 2000 according to the manufacturer’s protocol. An RNA-Quick Purification Kit (RN001; ES Science, Shanghai, China) was used to extract total RNA from the cells. PrimeScript RT reagent kits and SYBR Premix Ex Taq II kits (Takara Bio, Inc., Shiga, Japan) were used to detect mRNA expression, which was normalized to GAPDH.

### Malondialdehyde (MDA) detection

MDA is a lipid peroxidation marker^31^. The MDA level was detected using an MDA assay kit (Dojindo, Beijing, China). In brief, the standard curve was plotted according to the technical manual of the MDA kit. An MDA-TBA adduct is formed by the reaction of MDA in the sample and thiobarbituric acid (TBA), which can be quantified by fluorescence intensity using a microplate reader (Ex: 540 nm, Em: 590 nm). Then, the MDA concentration can be calculated based on the standard curve.

### Cell death assay

Cells were cultured in 6-well plates in the presence or absence of 5-fluorouracil (Selleck, Shanghai, China) and irradiation (2 Gy) treatment (CT/RT). The 5-fluorouracil was dissolved in dimethyl sulfoxide (DMSO) (Millipore Sigma). After a 48 h treatment, cells were collected and resuspended, then stained with propidium iodide (PI) (Dojindo) for 15 min, followed by flow cytometric analysis.

### Western blot analysis

Cells were lysed with RIPA buffer (SolarbioLife Science, Beijing, China) containing protease inhibitor cocktails and phosphatase inhibitors (NCM Biotech, Suzhou, China), and quantified using the BCA protein assay kit (Thermo Fisher Scientific). The protein samples were separated by SDS-PAGE and transferred onto PVDF membranes (Millipore Sigma). After incubation with 5% skimmed milk for 2 h, the membranes were incubated with the antibodies against the target proteins overnight at 4°C. The membranes were then thrice-washed with Tris-buffered saline containing 0.1% Tween 20 for 10 min each and incubated with HRP-conjugated secondary antibodies (1:5,000; Easy Bio, Inc., Seoul, South Korea) for 2 h at 25°C. Protein bands were detected using the ECL system. Primary antibodies against ALOX5 (1:1,000, #3289; Cell Signaling Technology, Danvers, MA, USA), NF-κB p65 (1:1,000, #8242; Cell Signaling Technology), phospho-NF-κB p65 [(Ser536) 1:1,000, #3033; Cell Signaling Technology], iNOS (1:1,000, ab178945; Abcam, Cambridge, UK), and α-Tubulin (1:10,000, 66031-1-Ig; Proteintech, Rosemont, IL, USA) were used. Three independent experiments were performed.

### Cell viability

Cells transfected with siRNA or siControl were seeded in 96-well plates. The cells were measured daily for 96 h using a Cell Counting Kit-8 (CCK-8; Dojindo Laboratories, Kumamoto, Japan). CCK-8 reagent (10 μL) was added to each well and incubated at 37°C for 1.5 h. Absorbance was measured at 450 nm.

### Statistical analysis

The HWE of the genotypes was determined using a chi-square (*χ*^2^) test. OS was calculated from the date of diagnosis until the time of death or the last follow-up visit. The associations between OS and genetic variations were evaluated using adjusted hazard ratios (HRs) with 95% confidence intervals (CIs), and calculated using Cox regression models and adjusted for gender, age, tumor stage, tumor grade, KPS, surgical procedure, and tumor location as covariates. Kaplan-Meier survival analysis was used to estimate the survival distributions, and the differences between groups were compared using the log-rank test. Haplo.stats (version 1.8.7) in the R package was used to estimate haplotype frequencies. Student’s *t*-test was used to determine the difference between the two groups. All statistical analyses were performed using R software (version 4.0.5) and SPSS (version 25). Statistical significance was set at *P* < 0.05.

### Bioinformatics analysis

The Genotype-Tissue Expression Project (GTEx database; http://www.gtexportal.org/) was used to demonstrate eQTL evidence between SNPs and gene expression levels. Regulomedb (https://www.regulomedb.org/regulome-search) and GWAVA (https://www.sanger.ac.uk/sanger/StatGen_Gwava) were used to prioritize the most likely functional variants from the SNPs that were significantly correlated with mRNA expression. ALOX5 co-expression analysis was performed using colorectal adenocarcinoma mRNA expression data [The Cancer Genome Atlas (TCGA), PanCancer Atlas] on the online website, cBioPortal (http://www.cbioportal.org/). Metascape (http://metascape.org/gp/index.html) was used to perform enrichment analysis for correlated genes with a *q* < 0.05 (Benjamini-Hochberg correction) and an *r* > 0.3 (Spearman’s correlation).

## Results

### Clinical characteristics of patients

The clinical characteristics of the patients are summarized in **Table 1**. At the time of the final analysis (April 1, 2021) the median follow-up duration was 114 months (range, 10–195 months). Among the 300 patients included in this study, 226 (75.3%) survived and 74 (24.7%) died, 126 (42.0%) had stage II tumors and 174 (58.0%) had stage III tumors, and 196 (65.3%) had tumors ≥ 5 cm from the anus and 104 (34.7%) had tumors < 5 cm from the anus. Dixon surgery was performed in 238 (79.3%) patients, whereas Mile’s surgery was performed in 62 (20.7%) patients. Additionally, 12 (4.0%) tumors were poorly graded, whereas 259 (86.3%) and 29 (9.7%) were moderately and well-graded, respectively. Moreover, 17 (5.7%) patients had a score of 70, and 151 (50.3%) and 132 (44.0%) patients had scores of 80 and 90–100, respectively. The median survival time was significantly shorter in patients with stage III rectal cancer than those with stage II cancer (*P* < 0.001). Moreover, patients with low KPS scores had shorter median survival times than patients with high KPS scores (*P* = 0.008). There were no significant differences between the OS of patients and other clinical characteristics (**Table 1**).

**Table 1:** Associations between clinical characteristics and overall survival

Characteristics	Total (%)	Alive (%)	Death (%)	*P*-value^†^
Gender				0.144
Male	181 (60.3)	131 (58.0)	50 (67.6)	
Female	119 (39.7)	95 (42.0)	24 (32.4)	
Age, years				0.114
≤ 56	159 (53.0)	126 (55.8)	33 (44.6)	
> 56	141 (47.0)	100 (44.2)	41 (55.4)	
Clinical stage				< 0.001
II	126 (42.0)	108 (47.8)	18 (24.3)	
III	174 (58.0)	118 (52.2)	56 (75.7)	
Tumor grade				0.071
Poor	12 (4.0)	8 (3.5)	4 (5.4)	
Moderate	259 (86.3)	200 (88.5)	59 (79.7)	
Well	29 (9.7)	18 (8.0)	11 (14.9)	
KPS				0.008
90–100	132 (44.0)	105 (46.5)	27 (36.5)	
80	151 (50.3)	113 (50.0)	38 (51.4)	
70	17 (5.7)	8 (3.5)	9 (12.1)	
Surgery				0.738
Dixon	238 (79.3)	180 (79.6)	58 (78.4)	
Mile’s	62 (20.7)	46 (20.4)	16 (21.6)	
Tumor location				0.660
Distance from anal < 5 cm	104 (34.7)	77 (34.1)	27 (36.5)	
Distance from anal ≥ 5 cm	196 (65.3)	149 (65.9)	47 (63.5)	

KPS, Karnofsky performance score. ^†^Log-rank test.

### Associations between individual SNPs and OS

Cox regression models adjusted for covariates (*P* < 0.05) revealed that among the 169 selected genetic variations (**Supplementary Table S2**), 16 (*CASP3* rs113420705 and rs4647700; *CASP7* rs12263370; *TRAILR2* rs11784599; *GSDME* rs2954558; *CASP4* rs571407, rs612987, rs623114, and rs543923; *ALOX5* rs702365, rs2242332, and rs4948673; *HO-1* rs17883419 and rs2071749; *GPX4* rs36207883; and *NRF2* rs73976300) were significantly associated with OS.

The genotype distributions of the significant SNPs in the patients are shown in **Table 2**. Five genetic variations (rs113420705, rs4647700, rs12263370, rs2954558, and rs17883419) were associated with a shorter OS. Compared to the *CASP3* rs113420705 CC genotype, the TT genotype had a significantly increased risk of mortality (adjusted HR = 2.41, 95% CI = 1.21–4.78; *P* = 0.012), while the CT genotype was not associated with an increased risk of mortality (adjusted HR = 1.46, 95% CI = 0.87–2.48; *P* = 0.154). Compared to the *CASP3* rs4647700 GAG-GAG genotype, the del genotype had a 3.53-fold increased risk of mortality (95% CI = 1.33–9.34; *P* = 0.011) while the GAG-del genotype was not associated with an increased risk of mortality (adjusted HR = 1.24, 95% CI = 0.73–2.11; *P* = 0.422). Because of the rarity of the homozygous variant genotype of *CASP7* rs12263370, *GSDME* rs2954558, and *HO-1* rs17883419 SNPs, we combined the homozygous variant genotype with the heterozygous genotype for analysis. Patients carrying at least one *CASP7* rs12263370 A allele (GA/AA genotype) had an adjusted HR of 1.69 (95% CI = 1.03–2.78; *P* = 0.039) compared to the GG genotype. Patients carrying at least one *GSDME* rs2954558 or *HO-1* rs17883419 T allele (CT/TT genotype) had an adjusted HR of 1.66 (95% CI = 1.03–2.67; *P* = 0.037) and 2.27 (95% CI = 1.40–3.69; *P* = 0.001) compared to the CC genotype, respectively.

**Table 2:** Relationships between 16 significant genetic variations and OS of rectal cancer patients in co-dominant and dominant models

Genotype	Alive (%)	Death (%)	Crude		Adjusted^†^	
			HR (95% CI)	*P*-value	HR (95% CI)	*P*-value
**rs113420705^‡^**						
CC	103 (46.2)	25 (34.2)	1.00 (reference)	–	1.00 (reference)	–
CT	95 (42.6)	34 (46.6)	1.41 (0.84–2.37)	0.191	1.46 (0.87–2.48)	0.154
TT	25 (11.2)	14 (19.2)	2.11 (1.09–4.06)	0.026	2.41 (1.21–4.78)	0.012
CT + TT	120 (53.8)	48 (65.8)	1.56 (0.96–2.53)	0.071	1.64 (1.00–2.68)	0.050
**rs4647700**						
GAG-GAG	170 (75.2)	49 (66.2)	1.00 (reference)	–	1.00 (reference)	–
GAG-del	54 (23.9)	20 (27.0)	1.28 (0.76–2.15)	0.355	1.24 (0.73–2.11)	0.422
del-del	2 (0.9)	5 (6.8)	4.11 (1.62–10.41)	0.003	3.53 (1.33–9.34)	0.011
GAG-del + del-del	56 (24.8)	25 (33.8)	1.48 (0.91–2.40)	0.110	1.42 (0.87–2.33)	0.162
**rs12263370^‡^**						
GG	167 (75.2)	49 (66.2)	1.00 (reference)	–	1.00 (reference)	–
GA	53 (23.9)	18 (24.3)	1.17 (0.68–2.02)	0.560	1.33 (0.76–2.33)	0.312
AA	2 (0.9)	7 (9.5)	5.68 (2.55–12.67)	< 0.001	4.92 (2.12–11.39)	< 0.001
GA + AA	55 (24.8)	25 (33.8)	1.51 (0.93–2.44)	0.096	1.69 (1.03–2.78)	0.039
**rs11784599**						
CC	156 (69.0)	59 (79.7)	1.00 (reference)	–	1.00 (reference)	–
CA	62 (27.4)	12 (16.2)	0.54 (0.29–1.00)	0.051	0.48 (0.25–0.91)	0.024
AA	8 (3.6)	3 (4.1)	0.90 (0.28–2.88)	0.864	0.64 (0.20–2.12)	0.468
CA + AA	70 (31.0)	15 (20.3)	0.59 (0.33–1.03)	0.065	0.51 (0.28–0.91)	0.022
**rs571407**						
TT	60 (26.5)	33 (44.6)	1.00 (reference)	–	1.00 (reference)	–
CT	122 (54.0)	31 (41.9)	0.50 (0.31–0.82)	0.006	0.46 (0.28–0.75)	0.002
CC	44 (19.5)	10 (13.5)	0.44 (0.22–0.89)	0.022	0.39 (0.19–0.82)	0.012
CT + CC	166 (73.5)	41 (55.4)	0.49 (0.31–0.77)	0.002	0.44 (0.28–0.70)	0.001
**rs612987**						
TT	45 (19.9)	23 (31.1)	1.00 (reference)	–	1.00 (reference)	–
CT	118 (52.2)	37 (50.0)	0.63 (0.37–1.06)	0.083	0.56 (0.33–0.96)	0.034
CC	63 (27.9)	14 (18.9)	0.46 (0.24–0.89)	0.022	0.45 (0.23–0.88)	0.020
CT + CC	181 (80.1)	51 (68.9)	0.57 (0.35–0.94)	0.026	0.52 (0.32–0.87)	0.013
**rs623114**						
AA	69 (30.5)	32 (43.2)	1.00 (reference)	–	1.00 (reference)	–
GA	114 (50.5)	31 (41.9)	0.57 (0.35–0.94)	0.028	0.52 (0.31–0.86)	0.010
GG	43 (19.0)	11 (14.9)	0.56 (0.28–1.11)	0.098	0.49 (0.24–0.99)	0.046
GA + GG	157 (69.5)	42 (56.8)	0.57 (0.36–0.90)	0.017	0.51 (0.32–0.82)	0.005
**rs543923^‡^**						
CC	135 (60.0)	53 (71.6)	1.00 (reference)	–	1.00 (reference)	–
CT	78 (34.7)	18 (24.3)	0.60 (0.35–1.03)	0.062	0.55 (0.32–0.94)	0.031
TT	12 (5.3)	3 (4.1)	0.59 (0.18–1.89)	0.374	0.52 (0.15–1.73)	0.286
CT + TT	90 (40.0)	21 (28.4)	0.60 (0.36–0.99)	0.047	0.54 (0.32–0.91)	0.022
**rs2954558**						
CC	158 (69.9)	44 (59.5)	1.00 (reference)	–	1.00 (reference)	–
CT	59 (26.1)	24 (32.4)	1.36 (0.83–2.24)	0.226	1.55 (0.93–2.59)	0.092
TT	9 (4.0)	6 (8.1)	2.11 (0.90–4.96)	0.086	2.27 (0.93–5.51)	0.071
CT + TT	68 (30.1)	30 (40.5)	1.46 (0.92–2.33)	0.107	1.66 (1.03–2.67)	0.037
**rs702365^‡^**						
GG	81 (36.0)	38 (51.4)	1.00 (reference)	–	1.00 (reference)	–
GC	105 (46.7)	28 (37.8)	0.60 (0.37–0.97)	0.038	0.60 (0.37–0.98)	0.041
CC	39 (17.3)	8 (10.8)	0.49 (0.23–1.04)	0.063	0.52 (0.24–1.12)	0.094
GC + CC	144 (64.0)	36 (48.6)	0.57 (0.36–0.90)	0.015	0.58 (0.37–0.92)	0.020
**rs2242332**						
CC	112 (49.6)	50 (67.6)	1.00 (reference)	–	1.00 (reference)	–
CT	96 (42.4)	20 (27.0)	0.50 (0.30–0.84)	0.009	0.47 (0.27–0.79)	0.005
TT	18 (8.0)	4 (5.4)	0.54 (0.20–1.50)	0.240	0.56 (0.20–1.59)	0.278
CT + TT	114 (50.4)	24 (32.4)	0.51 (0.31–0.82)	0.006	0.48 (0.29–0.79)	0.004
**rs4948673^‡^**						
TT	133 (59.1)	56 (75.7)	1.00 (reference)	–	1.00 (reference)	–
TA	83 (36.9)	16 (21.6)	0.49 (0.28–0.86)	0.013	0.49 (0.28–0.86)	0.013
AA	9 (4.0)	2 (2.7)	0.55 (0.13–2.25)	0.406	0.51 (0.12–2.14)	0.354
TA + AA	92 (40.9)	18 (24.3)	0.50 (0.29–0.85)	0.010	0.49 (0.28–0.84)	0.010
**rs36207883^‡^**						
GG	66 (30.4)	28 (40.0)	1.00 (reference)	–	1.00 (reference)	–
GA	111 (51.2)	36 (51.4)	0.85 (0.52–1.39)	0.513	0.66 (0.40–1.10)	0.114
AA	40 (18.4)	6 (8.6)	0.44 (0.18–1.07)	0.069	0.38 (0.16–0.93)	0.035
GA + AA	151 (69.6)	42 (60.0)	0.75 (0.46–1.21)	0.237	0.60 (0.37–0.98)	0.041
**rs17883419^‡^**						
CC	182 (80.9)	46 (63.0)	1.00 (reference)	–	1.00 (reference)	–
CT	38 (16.9)	23 (31.5)	2.09 (1.26–3.44)	0.004	2.29 (1.38–3.82)	0.001
TT	5 (2.2)	4 (5.5)	2.42 (0.87–6.74)	0.090	2.16 (0.76–6.14)	0.150
CT + TT	43 (19.1)	27 (37.0)	2.13 (1.32–3.43)	0.002	2.27 (1.40–3.69)	0.001
**rs2071749**						
GG	115 (50.9)	51 (68.9)	1.00 (reference)	–	1.00 (reference)	–
GA	96 (42.5)	21 (28.4)	0.56 (0.34–0.93)	0.024	0.57 (0.34–0.96)	0.036
AA	15 (6.6)	2 (2.7)	0.34 (0.08–1.41)	0.139	0.27 (0.06–1.12)	0.072
GA + AA	111 (49.1)	23 (31.1)	0.53 (0.32–0.87)	0.011	0.52 (0.32–0.87)	0.012
**rs73976300**						
CC	177 (78.3)	65 (87.8)	1.00 (reference)	–	1.00 (reference)	–
CT	47 (20.8)	8 (10.8)	0.50 (0.24–1.05)	0.067	0.40 (0.19–0.85)	0.017
TT	2 (0.9)	1 (1.4)	1.42 (0.20–10.22)	0.730	2.39 (0.31–18.53)	0.404
CT + TT	49 (21.7)	9 (12.2)	0.54 (0.27–1.09)	0.085	0.44 (0.21–0.91)	0.026

HR, hazard ratio; CI, confidence interval. ^†^Adjusted for gender, age, clinical stage, tumor grade, KPS, surgical procedure, and tumor location. ^‡^Some samples were not successfully genotyped.

**Figure 1:**
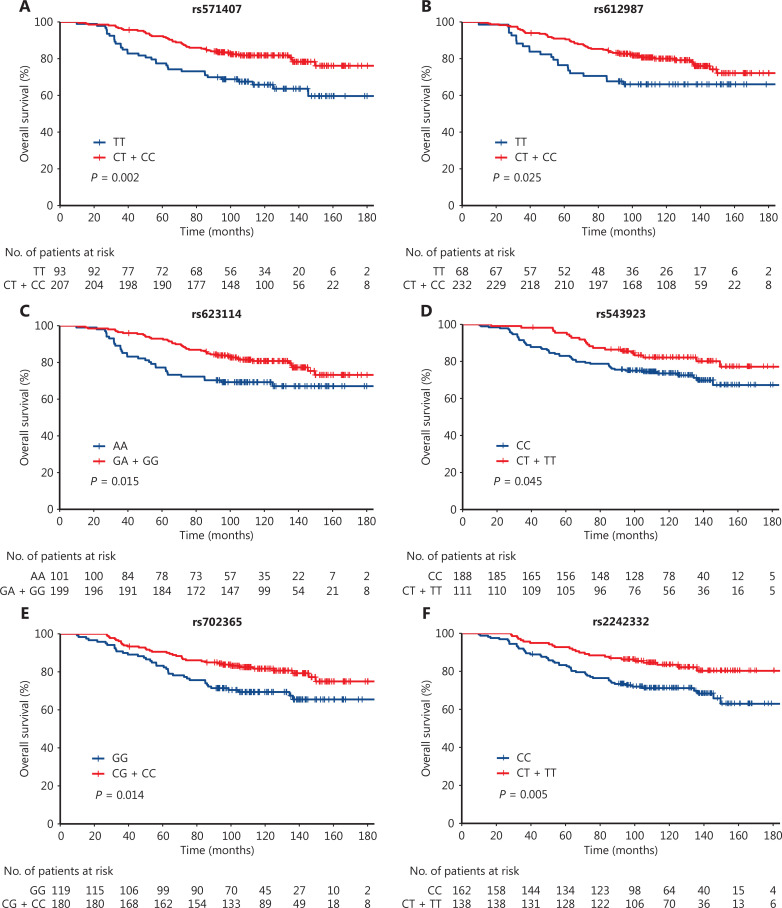

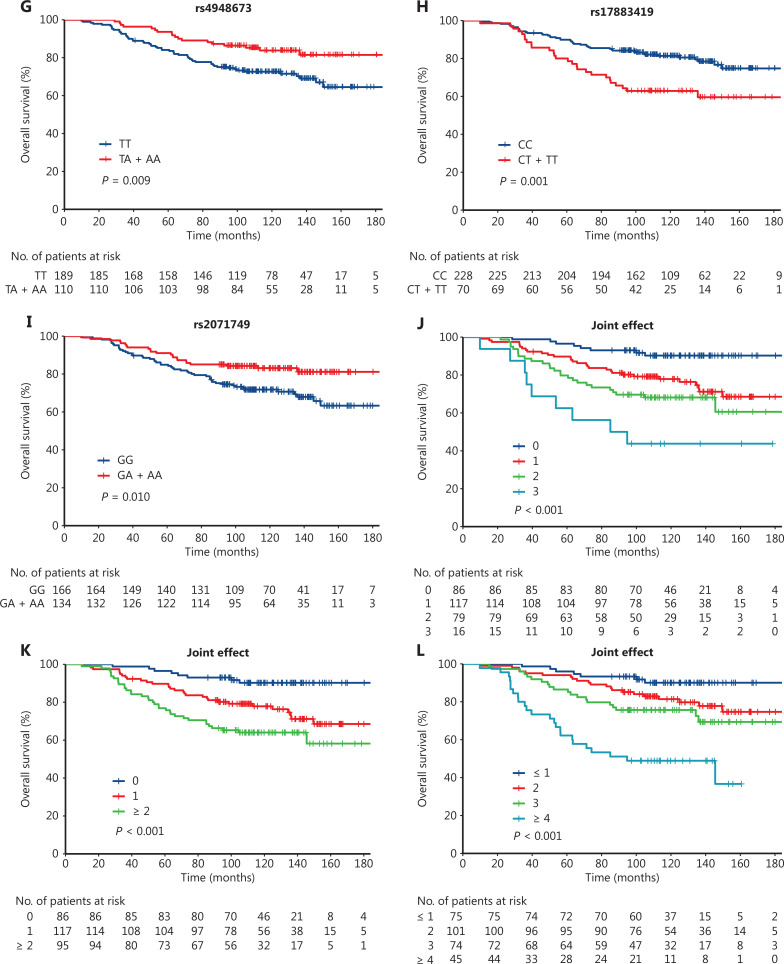
The Kaplan-Meier survival curves of rectal cancer patients treated with postoperative chemoradiotherapy by genotypes or different number of risk genotypes. (A) rs571407 (log-rank test, *P* = 0.002); (B) rs612987 (log-rank test, *P* = 0.025); (C) rs623114 (log-rank test, *P* = 0.015); (D) rs543923 (log-rank test, *P* = 0.045); (E) rs702365 (log-rank test, *P* = 0.014); (F) rs2242332 (log-rank test, *P* = 0.005); (G) rs4948673 (log-rank test, *P* = 0.009); (H) rs17883419 (log-rank test, *P* = 0.001); (I) rs2071749 (log-rank test, *P* = 0.010); (J) Four risk groups (log-rank test, *P* < 0.001) and (K) Three risk groups (log-rank test, *P* < 0.001) of joint effect of unfavorable genotypes (*CASP4* rs571407 TT, *ALOX5* rs2242332 CC, *HO-1* rs17883419 CT + TT). (L) Four risk groups (log-rank test, *P* < 0.001) of the unfavorable genotype joint effects (*CASP4* rs571407 TT, *ALOX5* rs2242332 CC, *HO-1* rs17883419 CT + TT, hsa-miR-4274 rs202195689 CCCCA-del + del-del, and *PMS1* rs5743030 GG).

In addition, we identified 11 SNPs (rs11784599, rs571407, rs612987, rs623114, rs543923, rs702365, rs2242332, rs4948673, rs36207883, rs2071749, and rs73976300) that were associated with a higher OS. The adjusted HRs were 0.51 (95% CI = 0.28–0.91; *P* = 0.022), 0.44 (95% CI = 0.28–0.70; *P* = 0.001), 0.52 (95% CI = 0.32–0.87; *P* = 0.013), 0.51 (95% CI = 0.32–0.82; *P* = 0.005), 0.54 (95% CI = 0.32–0.91; *P* = 0.022), 0.58 (95% CI = 0.37–0.92; *P* = 0.020), 0.48 (95% CI = 0.29–0.79; *P* = 0.004), 0.49 (95% CI = 0.28–0.84; *P* = 0.010), 0.60 (95% CI = 0.37–0.98; *P* = 0.041), 0.52 (95% CI = 0.32–0.87; *P* = 0.012), and 0.44 (95% CI = 0.21–0.91; *P* = 0.026) for the *TRAILR2* rs11784599 CA/AA, *CASP4* rs571407 CT/CC, *CASP4* rs612987 CT/CC, *CASP4* rs623114 GA/GG, *CASP4* rs543923 CT/TT, *ALOX5* rs702365 GC/CC, *ALOX5* rs2242332 CT/TT, *ALOX5* rs4948673 TA/AA, G*PX4* rs36207883 GA/AA, *HO-1* rs2071749 GA/AA, and *NRF2* rs73976300 CT/TT genotypes compared to the respective homozygous common genotype, respectively. SNPs that were significantly associated with OS in the Kaplan-Meier survival analysis are shown in **Figure 1A–1I** (log-rank *P* < 0.05) and included 4 SNPs of *CASP4* (rs571407, rs612987, rs623114, and rs543923; **Figure 1A–1D**), 3 SNPs of *ALOX5* (rs702365, rs2242332, and rs4948673; **Figure 1E–1G**), and 2 SNPs of *HO-1* (rs17883419 and rs2071749; **Figure 1H, 1I**). The other SNPs are shown in **Supplementary Figure S3A–S3G** (log-rank *P* > 0.05).

### Associations between combined SNPs and OS

We evaluated the additive effects of 3 SNPs (*CASP4* rs571407, *ALOX5* rs2242332, and *HO-1* rs17883419) that had the smallest *P*-value in the dominant model (**Table 2**). We selected the risk genotypes according to the results shown in **Table 2**, as follows: TT for rs571407; CC for rs2242332; and CT + TT for rs17883419. Compared to patients without the unfavorable genotypes, patients carrying 1, 2, or 3 unfavorable genotypes had an adjusted HR of 3.32 (95% CI = 1.51–7.27; *P* = 0.003), 4.66 (95% CI = 2.08–10.47; *P* < 0.001), or 10.62 (95% CI = 4.04–27.93; *P* < 0.001), respectively (**Table 3**), with a *P* value < 0.001 in the trend test, thus suggesting a significant cumulative effect of these SNPs. When patients with 2 or 3 risk genotypes were combined for analysis, the adjusted HR for mortality was 5.57 (95% CI = 2.55–12.16; *P* < 0.001) compared to patients without risk genotypes. Kaplan-Meier survival curves based on the number of unfavorable genotypes are shown in **Figure 1J, 1K**.

**Table 3:** Joint effect of three genetic variations in *CASP4*, *ALOX5*, and *HO-1* on OS of rectal cancer patients receiving postoperative CRT

Number of risk genotypes^‡^	Alive (%)	Death (%)	Crude		Adjusted^†^	
			HR (95% CI)	*P*-value	HR (95% CI)	*P*-value
0	78 (34.7)	8 (11.0)	1.00 (reference)	–	1.00 (reference)	–
1	87 (38.7)	30 (41.1)	2.94 (1.35–6.41)	0.007	3.32 (1.51–7.27)	0.003
2	53 (23.6)	26 (35.6)	4.21 (1.91–9.31)	< 0.001	4.66 (2.08–10.47)	< 0.001
3	7 (3.1)	9 (12.3)	8.77 (3.38–22.77)	< 0.001	10.62 (4.04–27.93)	< 0.001
*P*-value for trend				< 0.001		< 0.001
≥ 2 of risk genotypes	60 (26.7)	35 (47.9)	4.86 (2.25–10.48)	< 0.001	5.57 (2.55–12.16)	< 0.001

HR, hazard ratio; CI, confidence interval. ^†^Adjusted for gender, age, clinical stage, tumor grade, KPS, surgical procedure, and tumor location. ^‡^Risk genotypes were *CASP4* rs571407 TT, *ALOX5* rs2242332 CC, and *HO-1* rs17883419 CT + TT.

We have previously shown that hsa-miR-4274 rs202195689, *PMS1* rs5743030, rs4920657, and rs5743100 SNPs are significantly associated with OS (**Supplementary Table S3** and **Supplementary Figure S3H–S3K**)^26,27^. In the present study, we jointly analyzed these two SNPs (rs202195689 and rs5743030) with the three above-mentioned SNPs and showed that the HR for mortality increased as the number of risk genotypes increased (*P*_trend_ < 0.001; **Supplementary Table S4**). Compared to patients without the risk genotype or carrying 1 risk genotype, the HRs of mortality for patients with 2, 3, or ≥ 4 risk genotypes were 2.58 (95% CI = 1.08–6.13; *P* = 0.033), 3.88 (95% CI = 1.63–9.22; *P* = 0.002), and 9.68 (95% CI = 4.08–22.97; *P* < 0.001), respectively. The Kaplan-Meier survival curves of this joint analysis are shown in **Figure 1L** and **Supplementary Figure S3L**.

### Haplotype analyses of SNPs

Haplotypes were estimated using 4 *CASP4* SNPs (rs571407, rs612987, rs623114, and rs543923; **Table 4**). Compared to patients with the haplotype, CTAT (H1), patients with the haplotype, CCGC (H2) or TCGC (H3), had significantly longer OS times with adjusted HRs for mortality of 0.52 (95% CI = 0.30–0.92; *P* = 0.026) and 0.50 (95% CI = 0.27–0.92; *P* = 0.027), respectively. No significant associations were observed for the other three haplotypes (TCAT, CTAC, and CTGC). We also analyzed the associations between the *ALOX5* haplotypes (rs702365, rs2242332, and rs4948673) and OS. The results (**Table 5**) showed that although the haplotypes, CCT(H3) or CTT (H4), were not significantly associated with a decreased risk of mortality (adjusted HR = 0.87, 95% CI = 0.44–1.72; *P* = 0.692 and HR = 0.67, 95% CI = 0.30–1.52; *P* = 0.336, respectively), the haplotype, CTA (H2), was significantly associated with a decreased risk of mortality, with an adjusted HR of 0.50 (95% CI = 0.28–0.88; *P* = 0.018) compared to the haplotype, GCT (H1).

**Table 4:** Relationships between *CASP4* SNP haplotypes and OS of rectal cancer patients with postoperative CRT

Haplotypes	A	B	C	D	Total (%)	Alive (%)	Death (%)	Crude		Adjusted^†^	
								HR (95% CI)	*P*-value	HR (95% CI)	*P*-value
H1	C	T	A	T	41.5	37.5	53.2	1.00 (reference)	–	1.00 (reference)	–
H2	C	C	G	C	19.5	20.1	17.6	0.58 (0.34–0.98)	0.043	0.52 (0.30–0.92)	0.026
H3	T	C	G	C	16.8	17.6	14.1	0.54 (0.30–0.95)	0.033	0.50 (0.27–0.92)	0.027
H4	T	C	A	T	4.3	5.1	2.1	0.27 (0.08–0.97)	0.046	0.27 (0.07–1.03)	0.057
H5	C	T	A	C	9.5	9.9	8.2	0.53 (0.27–1.07)	0.077	0.57 (0.27–1.21)	0.146
H6	C	T	G	C	5.1	5.8	3.4	0.45 (0.16–1.23)	0.121	0.47 (0.16–1.37)	0.169
H7	C	C	A	T	1.6	2.2	0.0	–	–	–	–
Rare^‡^	–	–	–	–	–	–	–	–	–	–	–

HR, hazard ratio; CI, confidence interval; A, rs543923; B, rs571407; C, rs623114; D, rs612987. ^†^Adjusted for gender, age, clinical stage, tumor grade, KPS, surgical procedure, and tumor location. ^‡^Rare: Haplotypes with frequencies < 1%.

**Table 5:** Relationships between *ALOX5* SNP haplotypes and OS of rectal cancer patients with postoperative CRT

Haplotypes	A	B	C	Total (%)	Alive (%)	Death (%)	Crude		Adjusted^†^	
							HR (95% CI)	*P*-value	HR (95% CI)	*P*-value
H1	G	C	T	61.5	58.9	69.6	1.00 (reference)	–	1.00 (reference)	–
H2	C	T	A	19.4	21.6	12.8	0.50 (0.29–0.87)	0.014	0.50 (0.28–0.88)	0.018
H3	C	C	T	11.4	11.6	10.8	0.80 (0.43–1.50)	0.490	0.87 (0.44–1.72)	0.692
H4	C	T	T	6.9	7.2	6.1	0.74 (0.34–1.58)	0.436	0.67 (0.30–1.52)	0.336
Rare^‡^	–	–	–	–	–	–	–	–	–	–

HR, hazard ratio; CI, confidence interval; A, rs702365; B, rs2242332; C, rs4948673. ^†^Adjusted for gender, age, clinical stage, tumor grade, KPS, surgical procedure, and tumor location. ^‡^Rare: Haplotypes with frequencies < 1%.

### Functional relevance of the ALOX5 rs702365 variant

**Figure 2:**
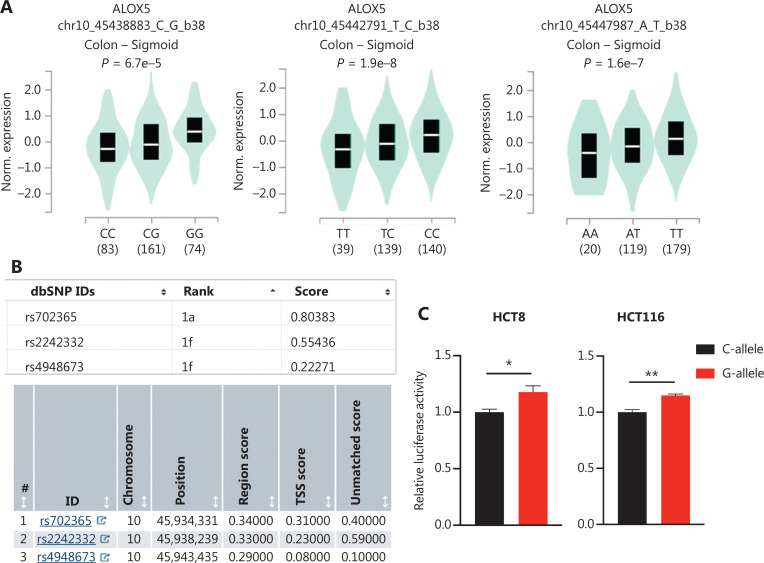

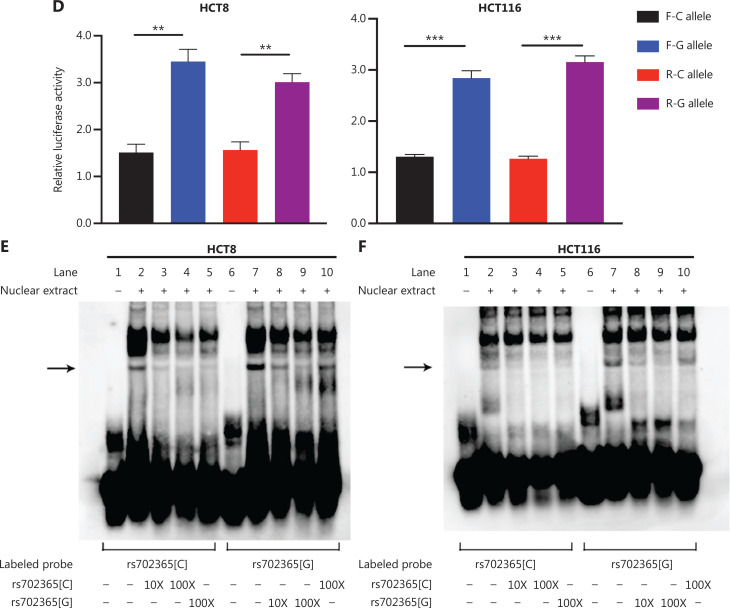
rs702365 variant at *ALOX5* intron region functions in an allele-specific manner. (A) eQTL analysis of *ALOX5* rs702365 (left), rs2242332 (middle), and rs4948673 (right) variants and associations with mRNA expression from GTEx in normal colon tissue. (B) Functional annotation of three SNPs in RegulomeDB (up) and GWAVA database (down). (C) Relative luciferase activity of the reporter gene with reconstructed plasmid containing rs702365[C]- or [G]-allele in constructs containing SV40 in HCT8 (left) and HCT116 (right) cells. (D) Relative luciferase activity of the reporter gene with reconstructed plasmids containing rs702365[C]- or [G]-allele in constructs containing *ALOX5* promoter in HCT8 (left) and HCT116 (right) cells. Relative luciferase activities are shown as fold changes relative to luciferase expression in cells transfected with empty vector. All constructs were co-transfected with pRL-TK to standardize transfection efficiency. Data represent the mean ± SEM from three independent experiments, each with three replicates. *P*-values were obtained using Student’s *t*-test. (E, F) EMSAs with biotin-labeled probes containing rs702365-[C] or -[G] allele in HCT8 (E) and HCT116 (F) cells. Arrow, allele-specific bands that interact with nuclear extracts. 10× and 100× indicate 10-fold and 100-fold excess amounts of an unlabeled probe compared with the amount of the labeled probe, respectively. “−” and “+” represent not added and added, respectively. *P < 0.05, **P < 0.01, ***P < 0.001.

We investigated the correlation between each SNP and the corresponding candidate gene mRNA expression in normal colon tissues by eQTL analysis using data from the GTEx project. Only three SNPs (rs702365, rs2242332, and rs4948673) were significantly correlated with *ALOX5* mRNA expression in the additive model (**Figure 2A**). Functional annotation methods (RegulomeDB and GWAVA) prioritized rs702365 as a potential functional variant, indicating a potential functional effect of rs702365 (**Figure 2B**). Moreover, analysis using the ENCODE database indicated that the region containing rs702365 [G/C] is hypersensitive to DNase and rich in H3K4Me1 and H3K27ac (**Supplementary Figure S2C**). Information from the RegulomeDB database also showed that this region might function as a regulatory element (**Supplementary Figure S2D**). We then performed dual-luciferase reporter assays with pGL4.10-SV40 vector containing allele-different 540-bp fragment with rs702365 as the center and found that the construct containing the rs702365 [G] allele had higher enhancer activity than the rs702365 [C] allele (**Figure 2C**). We then transfected the same plasmid, but replaced SV40 with *ALOX5* promoter to determine whether the substitution in rs702365 from [G] to [C] affected *ALOX5* promoter activity in HCT8 and HCT116 cells (**Figure 2D**). The plasmid containing the rs702365 [G] allele, regardless of the 5′ or 3′ direction in the construct, exhibited significantly higher reporter activity than those containing the rs702365 [C] allele, suggesting that rs702365 might have allele-specific enhancer activity.

In addition, we performed EMSA to determine whether the substitution from rs702365 [G] to [C] affected the binding of any nuclear proteins. The rs702365 [G] allele was preferentially bound to nuclear extracts compared to the rs702365 [C] allele in HCT8 and HCT116 cells [**Figure 2E, 2F** (lane 2 *vs.* lane 7)]. Additionally, competition assays showed that the DNA-protein complex formed by the interactions between rs702365 [G]-containing DNA and nuclear extracts was abolished when we added 100-fold excess unlabeled rs702365 [G] probe [**Figure 2E, 2F** (lane 9)], but not when the rs702365 [C] probe (**Figure 2E, 2F**, lane 10) was added to the reaction mixture. These findings suggested that the interaction between rs702365 [G]-containing DNA and nuclear proteins is sequence-specific.

### ALOX5 may promote proliferation *via* an inflammatory response in colon cancer cells

**Figure 3:**
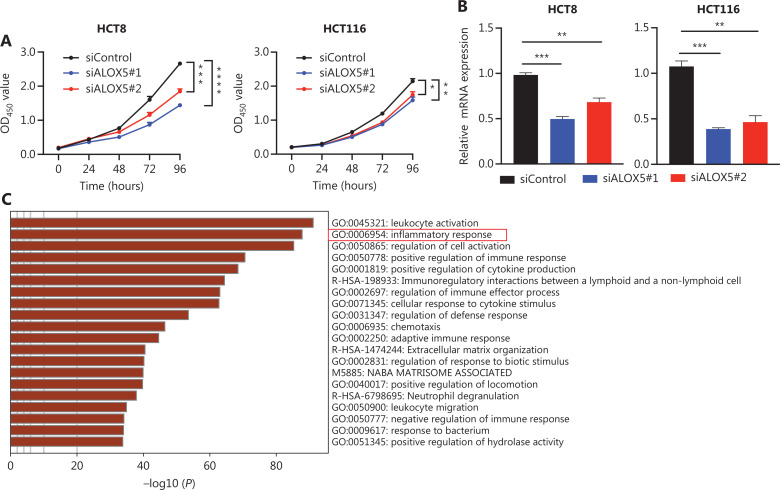

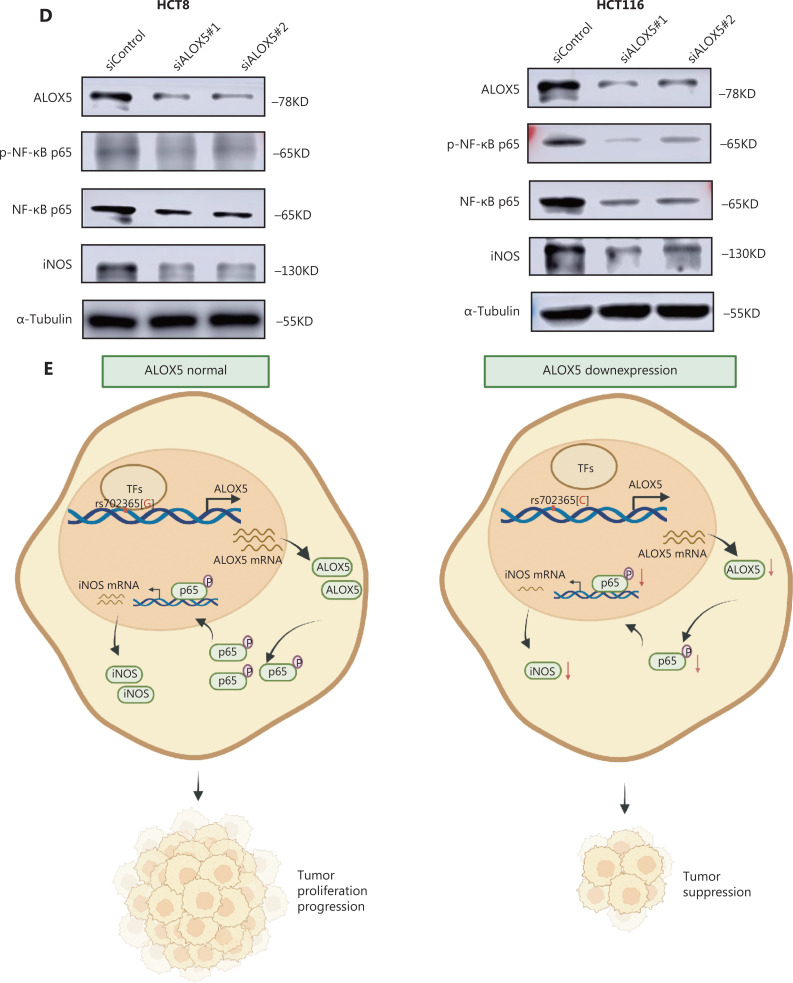
*ALOX5* may promote proliferation of colon cancer cells by inducing an inflammatory response. (A) The effects of *ALOX5* knockdown on the proliferation of HCT8 (left) and HCT116 (right) cells. (B) The expression of *ALOX5* in HCT8 (left) and HCT116 (right) cells detected by RT-qPCR after transfection with siControl or siALOX5. GAPDH was used as the reference gene. (C) Enrichment analysis results of *ALOX5* co-expression genes in Metascape. (D) The expression of inflammation-related protein in HCT8 (up) and HCT116 (down) cells transfected with siControl or siALOX5. (E) Schematic of the mechanism by which *ALOX5* rs702365 [G] > [C] substitution functions as a tumor suppressor in CRC. *ALOX5* rs702365 [G] > [C] change directly decreased ALOX5 expression. The down-expression of ALOX5 led to decreased expression of p-p65 and iNOS, which contributed to the inhibition of proliferation of colon cancer cells. Data represent the mean ± SEM based on three independent experiments. *P < 0.05, **P < 0.01, ***P < 0.001, ****P < 0.0001.

It has been reported that activation of ALOX5 leads to lipid peroxidation and oxidative cell death^32,33^. We verified that finding in colon cancer cell lines and found that lipid peroxide and the cell death percentage were reduced in HCT8 and HCT116 cells treated with CT/RT after *ALOX5* knockdown by siRNAs (**Supplementary Figure S4A–S4D**). To further determine the reason why decreased expression of ALOX5 can improve the prognosis of rectal cancer patients, we continued to transfect *ALOX5* siRNAs into HCT8 and HCT116 cells to investigate whether ALOX5 plays a role in CRC tumorigenesis. Additionally, a CCK-8 assay was performed to determine whether ALOX5 promotes the growth of HCT8 and HCT116 cells. Cell proliferation was significantly inhibited in the si-ALOX5 group (**Figure 3A**). RT-qPCR was used to confirm the efficiency of si-ALOX5 interference with ALOX5 in HCT8 and HCT116 cells (**Figure 3B**).

To investigate the possible mechanism underlying the effect of *ALOX5*, a total of 1,895 genes co-expressed with *ALOX5* that were identified by the cBioPortal website using CRC data from the TCGA database, were applied for Kyoto Encyclopedia of Genes and Genomes (KEGG) pathway enrichment analysis and Gene Ontology (GO) functional annotation analysis online on the Metascape website. Enrichment analysis revealed that these genes were mainly enriched in inflammatory and immune responses (**Figure 3C**).

Inflammation plays a key role in the occurrence and development of CRC^34,35^. Therefore, we determined the expression of the pro-inflammatory mediator, inducible nitric oxide synthase (iNOS), and the inflammatory gene-related transcription factor, NF-κB^36–38^. The expression of p-NF-κB p65 was decreased in the ALOX5 knockdown group compared to the control group (**Figure 3D**). These results suggested that *ALOX5* promotes CRC progression by mediating the inflammatory response. In summary, our findings indicated that the *ALOX5* rs702365 [G] > [C] change has a tumor suppressive role in CRC by decreasing ALOX5 transcription (**Figure 3E**).

## Discussion

CRC survival exhibits gender and geographic differences^39^. In recent years there have been numerous studies that have attempted to identify prognostic markers for cancer treatment^40,41^. Radiotherapy and chemotherapy are standard cancer treatment methods; however, patients with cancer often develop resistance to these treatments. Therefore, it is very important to find genetic molecular markers that will facilitate personalized treatment. Recently, the roles of apoptosis, pyroptosis, and ferroptosis in radiosensitivity and chemosensitivity have been reported^15–18,42–48^.

In this study we investigated the associations between 169 genetic variations in 40 genes involved in apoptosis, pyroptosis, and ferroptosis with the OS of locally advanced rectal cancer patients receiving postoperative CRT and identified 16 significantly associated genetic variations. Among the genetic variations, 5 (*CASP3* rs113420705 and rs4647700, *CASP7* rs12263370, *GSDME* rs2954558, and *HO-1 rs17883419*) were associated with a shorter OS and 11 (*TRAILR2* rs11784599; *CASP4* rs571407, rs612987, rs623114, and rs543923; *ALOX5* rs702365, rs2242332, and rs4948673; *GPX4* rs36207883; *HO-1* rs2071749; and *NRF2* rs73976300) were associated with a longer OS. Three SNPs (*CASP4* rs571407, *ALOX5* rs2242332, and *HO-1* rs17883419) exhibited cumulative effects, which increased the risk of mortality with an increased number of high-risk genotypes. Additionally, the genetic variations in *CASP4* and *ALOX5* haplotypes were associated with a higher OS. We also revealed, using functional annotation and prediction through GTEx, RegulomeDB, and GWAVA, that rs702365 was likely a functional SNP. Further biochemical experiments suggested that the rs702365 variant may regulate *ALOX5* expression *via* a long-range regulatory mechanism and influence the proliferation of colon cancer cells.

CASP3 has important roles in apoptosis and pyroptosis. CASP3 is a downstream effector of the caspase cascades in the apoptosis pathway and cleaves gasdermin E (GSDME) to induce pyroptosis^13^. The *CASP3* rs1049253 TT genotype is significantly associated with longer second primary malignancy-free survival compared to the TC/CC genotypes^49^. A *CASP3* rs113420705 C > T change decreases the risk of lung cancer^50^. Additionally, CASP4 triggers pyroptosis by cleaving gasdermin D^51^ and may act as a tumor suppressor in gastric cancer and esophageal squamous cell carcinoma^52,53^. In contrast, a recent study has shown that high CASP4 expression is associated with poor survival and decreased sensitivity to chemotherapy in clear cell carcinoma^54^. In the present study we demonstrated that 4 *CASP4* SNPs (rs571407, rs612987, rs623114, and rs543923) were associated with a longer OS time, and the haplotypes, CCGC (H2) and TCGC (H3), were associated with a reduced risk of mortality compared to the haplotype, CTAT (H1), in patients with rectal cancer receiving postoperative CRT. Therefore, further investigation of these SNPs in *CASP4* is required to determine the function in rectal cancer.

The main mechanism underlying the ferroptosis effect is that given the function of iron or lipoxygenases (LOXs), the highly expressed polyunsaturated fatty acids (PUFAs) on the cell membrane are catalyzed to generate lipid ROS, which induce cell death^21,55^. Among the six arachidonate LOXs identified in humans, ALOX5 has an important role in leukotriene (LTs) synthesis^56^. Activation of ALOX5 occurs selectively in ferroptosis-sensitive cells, leading to lipid peroxidation and oxidative cell death^33^. Additionally, ALOX5 activity is critical for the inflammatory response. ALOX5 is required for the recruitment of eosinophils in the abdominal cavity and blocking ALOX5 inhibits inflammatory and immune responses^57^. ALOX5 participates in the biosynthesis of LTs, which are important inflammatory mediators causing inflammatory symptoms that include the accumulation of leukocytes. Several studies have been conducted on the association between *ALOX5* and cancer susceptibility or survival^58–61^. *ALOX5* rs2115819 and rs12264801 are associated with poor survival in ovarian cancer^59^. A variable nucleotide tandem repeat polymorphism in *ALOX5* promoter is significantly associated with a lower risk of rectal cancer^61^. In addition, there are a few studies on the function of ALOX5 in CRC^62,63^. ALOX5 is upregulated in colon cancer, and its inhibition suppresses CRC progression through the PI3K/AKT pathway^63,64^. In the present study we showed that the rs702365 [C] allele altered the transcriptional activity, which subsequently decreased *ALOX5* expression, leading to the inhibition of lipid peroxidation and cell death after CT/RT treatment; however, the rs702365 [C] allele also inhibits the inflammatory response and proliferation of colon cancer cells, thereby having a protective role.

In recent years a few studies have been published on the relationship between genetic variations and the risk of cancer or patient survival. We have previously reported that the rs202195689 SNP in the microRNA seed region and rs5743030, rs4920657, and rs5743100 SNPs in *PMS1* are associated with OS in patients with rectal cancer, indicating that these variants may predict the prognosis of patients receiving postoperative CRT^26,27^. Notably, the present study is the first study to comprehensively evaluate the effect of genetic variations in genes related to the three well-known RCD pathways on the prognosis of patients with rectal cancer receiving postoperative CRT.

There were a few limitations to the present study. First, all the subjects involved in this study were recruited from the same hospital, thus selection biases cannot be ignored. Second, the sample size was not large enough.

## Conclusions

In summary, we identified 16 genetic variations in genes related to apoptosis, pyroptosis, and ferroptosis that were associated with the prognosis of patients with rectal cancer receiving postoperative CRT. Our results suggest that these SNPs may serve as potential prognostic biomarkers for patients with rectal cancer. We demonstrated that *ALOX5* rs702365 [G] > [C] substitution leads to decreased *ALOX5* expression. Moreover, preliminary *in vitro* experiments suggested that reduced ALOX5 expression decreased the proliferative ability of colon cancer cells by inhibiting an inflammatory response, ultimately suppressing the development of colon cancer, which should be thoroughly tested by more functional assays. Nevertheless, our results should be further validated by a large-scale study, and the functional molecular mechanisms of the other SNPs also require further investigation.
