# Supplementary material for: Genetic polymorphisms in genes regulating cell death and prognosis of patients with rectal cancer receiving postoperative chemoradiotherapy
Source: Cancer Biol Med. 2023 May 4;20(4):297–316. doi: 10.20892/j.issn.2095-3941.2022.0711 (PMC10157810; doi:10.20892/j.issn.2095-3941.2022.0711)
Supplement: Supplementary file 1 [file cbm-20-297-s001.pdf]

Supplementary materials

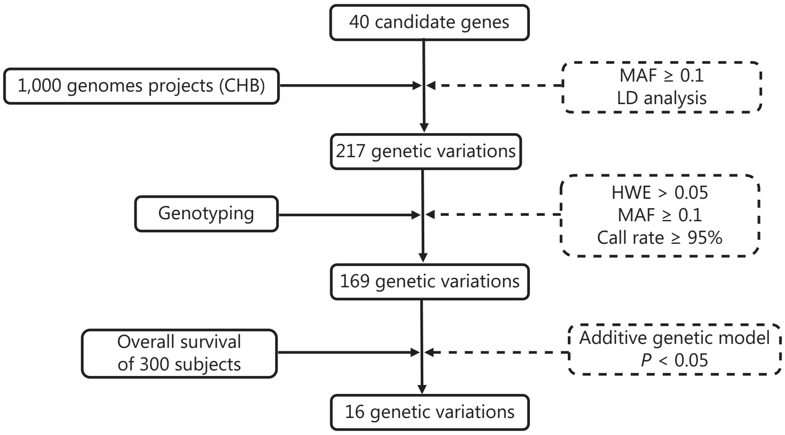

Figure S1 Flow chart for selecting genetic variations in the apoptosis, pyroptosis, and ferroptosis pathway genes. CHB, Chinese Han Beijing population; MAF, minor allele frequency; LD, linkage disequilibrium; HWE, Hardy-Weinberg equilibrium.

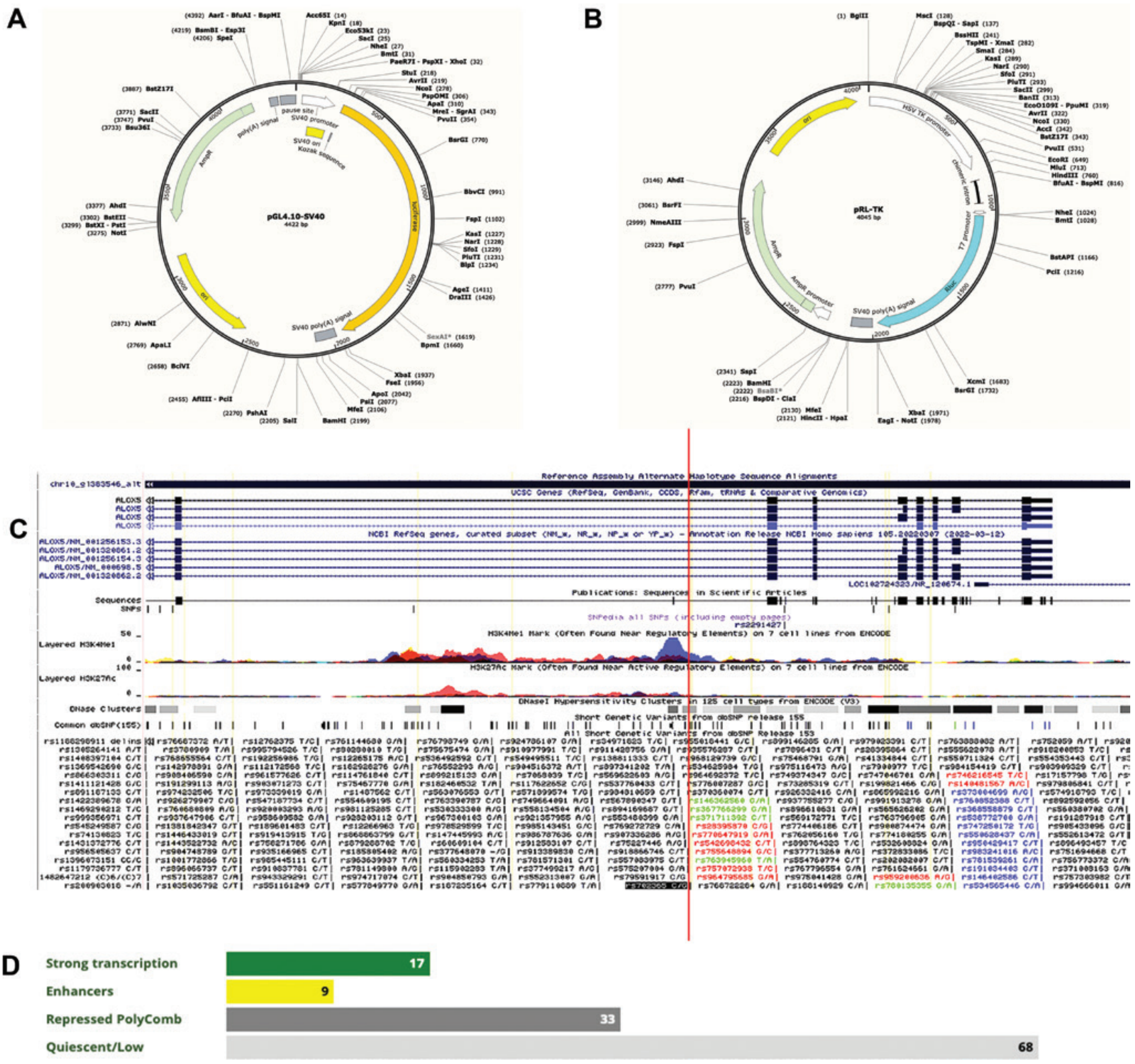

Figure S2 The maps of pGL4.10-SV40 vector and pRL-TK plasmid and functional annotation of region around rs702365. (A) The pGL4.10-SV40 vector map. (B) The pRL-TK plasmid map. (C) Annotation of intronic regions containing rs702365 using the ENCODE database. Colored histograms denote histone modification markers (H3K4Me1 and H3K27ac) in 7 human cell types. The darkness of DNase hypersensitive peaks represents the maximum signal strength observed in 125 cell lines. The red line indicates the location of rs702365. (D) Chromatin state data surrounding rs702365 in RegulomeDB.

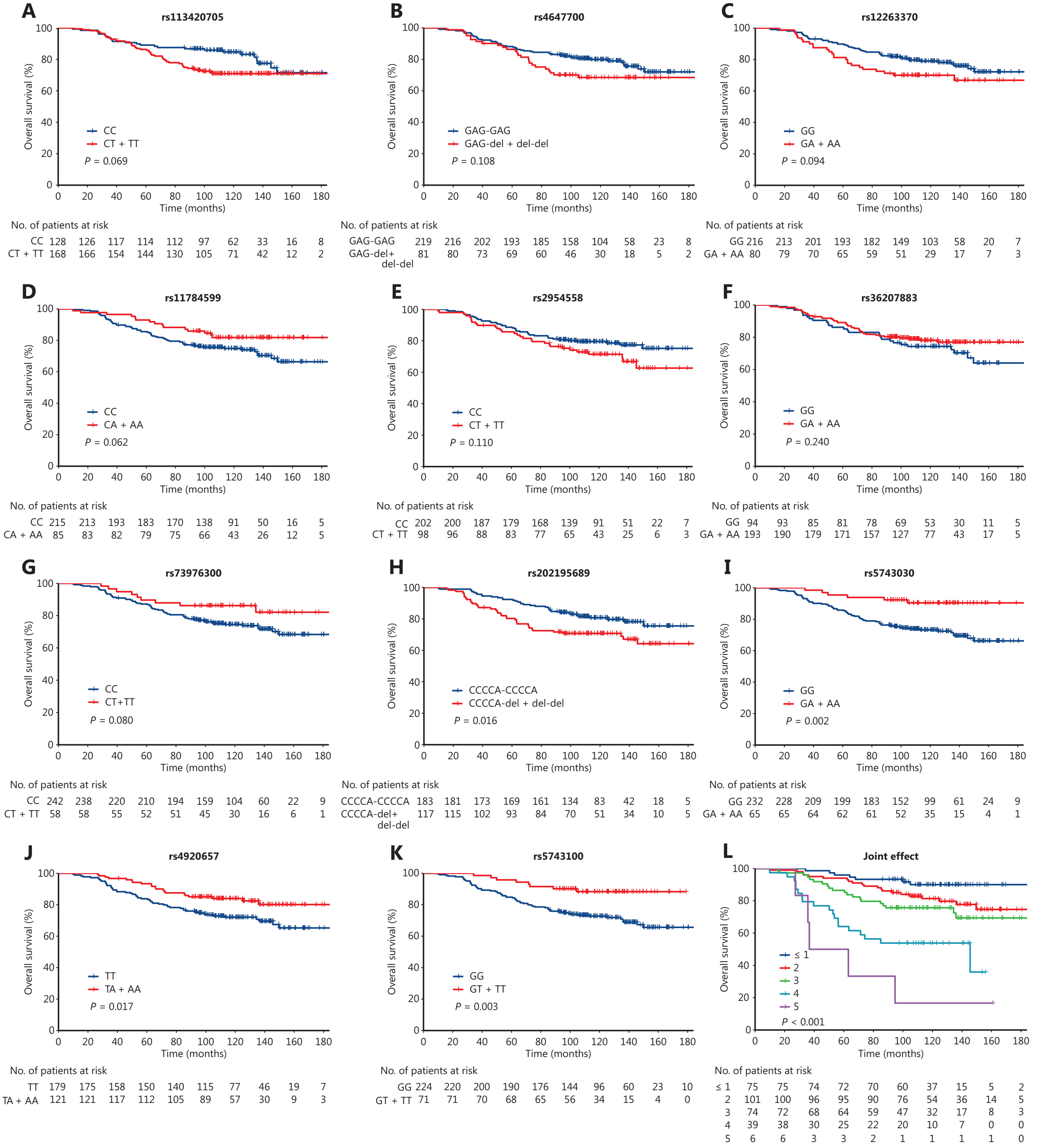

Figure S3 The Kaplan-Meier survival curves of rectal cancer patients treated with postoperative chemoradiotherapy by genotypes. (A) rs113420705 (log-rank test, P = 0.069); (B) rs4647700 (log-rank test, P = 0.108); (C) rs12263370 (log-rank test, P = 0.094); (D) rs11784599 (log-rank test, P = 0.062); (E) rs2954558 (log-rank test, P = 0.110); (F) rs36207883 (log-rank test, P = 0.240); (G) rs73976300 (log-rank test, P = 0.080); (H) rs202195689 (log-rank test, P = 0.016); (I) rs5743030 (log-rank test, P = 0.002); (J) rs4920657 (log-rank test, P = 0.017); (K) rs5743100 (log-rank test, P = 0.003); (L) Five risk groups (log-rank test, P < 0.001) of the joint effect of unfavorable genotypes (*CASP4* rs571407 TT, *ALOX5* rs2242332 CC, *HO-1* rs17883419 CT + TT, hsa-miR-4274 rs202195689 CCCCA-del + del-del, and *PMS1* rs5743030 GG).

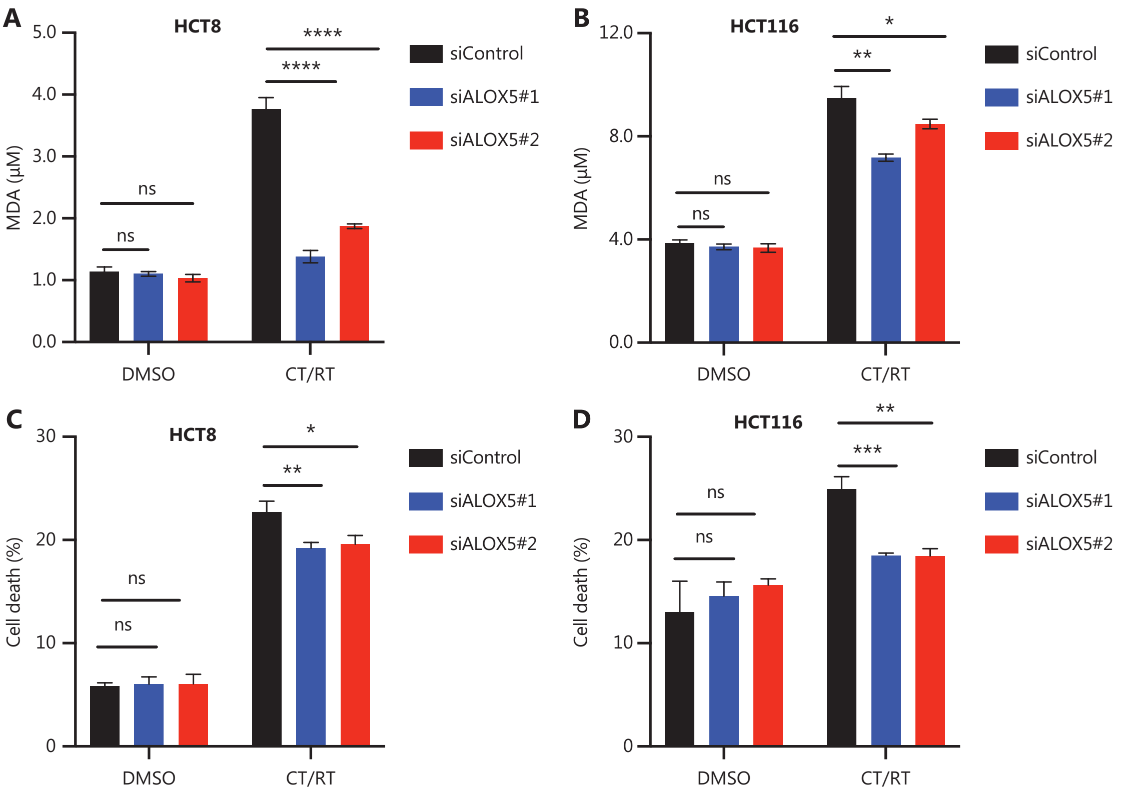

Figure S4 ALOX5-mediated lipid peroxidation and cell death. (A, B) HCT8 (A) and HCT116 (B) cells transfected with siControl or ALOX5 siRNA were treated with DMSO or CT/RT, followed by detecting the concentration of MDA. (C, D) HCT8 (C) and HCT116 (D) cells transfected with siControl or ALOX5 siRNA were cultured with DMSO or CT/RT, followed by analyzing of cell death. Data are presented as the mean ± SD. These assays were performed three times. DMSO: dimethyl sulfoxide; CT/RT: 5-fluorouracil (dissolved in DMSO) and irradiation treatment; $^{*}P < 0.05$, $^{**}P < 0.01$, $^{***}P < 0.001$, $^{****}P < 0.0001$.

Table S1 Sequences of primers and promoter used for vector construction, probes of EMSA, RT-qPCR and ALOX5 siRNAs

Expression vector construction	Primer sequence (5′→3′)
pGL4.10-rs702365[C]	Forward: CTAGCTAGCTGGGGACATGTCAGAAAGTGTCTCA
	Reverse: GATCGATCGACATGAAACCAATCTAAAACTATTA
ALOX5 promoter	Forward: CTCGAGATAGCACTGCGGTAATCAAG
	Reverse: AAGCTTGGACCAGACTGAAG
Promoter	Sequence (5′→3′)
ALOX5 promoter	ATAGCACTGCGGTAATCAAGCAGTGTGGCACTGTATAGCATGTACATTACAGATCAGTGGACTAGAATCA
	ATGTCCAGAAATAAACCGTTATGTTTATAATGAATTACTTTTTAATAAGGTGTCAAGACAACGCAATGGGA
	AAAGAATAATGAATTCAACAAATGATGCATGGACAACCGGACATGCACATGCAACACAATGAATTTGAAT
	TCTTCTATCGCTCCATGCATAAAAACTAACTCAAAATGGGTCACGGATGTAAATGAAAAGCTAAAACTATA
	ATAATCCTAGAGGAAAACCTAGGAGTAAATCTTTAAGATGTTATTGTAGGCAGTGGTTTCTCAGATAGGA
	CCCCAAAATCACAAGCGACAAAAGAAATTGGACTTAAAGTTAAATACTTTTGTGCTTCAAACATCATCAA
	GAAAGTGAAAACACAACCCGCAGAAGCAATAAAAATGTCTGTAAGTCATGTATCCGATTAGAGACTTCTA
	TCCAGGATATATAAATAATGCAATTCAATGATAAAAAAGATAAATAGCCCAGTTTTCCAAAGAGTCAAGCA
	TCTGAATATACATCTCTCCAAAAATATACAGATATCCAACAAGCATGTGAAAAGATGTTCAAAGCCATTTGC
	CAGGTGCACAAACCCAAGACAGTATGAGGAGATGCTACAGGGACTCTGCTGCTTCACAGACATGAAGCG
	TTGGTGAGAATGTAGGCAGCCGCCTTTGGGGACTTCACATCCCCGCCGCCCCACGCACGGTGAGCTAGT
	GTTTAAACTTAGCCGAGATCAATACACGCGACTGTGTGCCCGTCAGACCCTGCGCTGCCGGCGGGGCTG
	GGAGAGGCGGGCGCCAGGAGTGGGCGGGAACCTGGGGGTCAGGCCCCAGCCGCGGGAAGCCGCCC
	AGGAGCGCGCGAAACCTTCTCCACACCCTTCCAGGCATTTGCCCGCCGCGATTCAGAGAGCCGACCCGT
	GACCCCTGGCCTCCCCTAGACAGCCCCGCATGTCCAGATGTGCCGTCCCGCCTGCCTCCCGCGACCACTG
	GCCATCTCTGGGCCTGGGCGCGGTCTCGGCGCCCGCCTGCCCCCGCCAGGAGCCGCAGGTCCAGCCAG
	TGAAGAAGCCCGCGCTGAAGGAGCCTCTGTGCTCCAGAATCCATCCTCAGTATCAGCGCTGGGGTGGCC
	TCCTCCAGGAAGCCCTTCTGATTCTCTCATGGGTCGCTCTTCCTCTGCAGACTCCCGGAGCACCCCTGCTC
	CAAGTACCGCAAGTGGCACTGAGAACTTGGGGAGAGCAGAGGCTGTGCCTAGATTTGTAGGGAGTCCC
	CGCAGCTCCACCCCAGGGCCTACAGGAGCCTGGCCTTGGGCGAAGCCGAGGCAGGCAGGCAGGGCAA
	AGGGTGGAAGCAATTCAGGAGAGAACGAGTGAACGAATGGATGAGGGGTGGCAGCCGAGGTTGCCCC
	AGTCCCCTGGCTGCAGGAACAGACACCTCGCTGAGGAGAGACCCAGGAGCGAGGCCCCTGCCCCGCCC
	GAGGCGAGGTCCCGCCCAGTCGGCGCCGCGTGAAGAGTGGGAGAGAAGTACTGCGGGGGCGGGGGC
	GGGGGCGGGGGCGGGGGCGGGGGCAGCCGGGAGCCTGGAGCCAGACCGGGGCGGGGCCGGGACC
	GGGGCCAGGGACCAGTGGTGGGAGGAGGCTGCGGCGCTAGATGCGGACACCTGGACCGCCGCGCCG
	AGGCTCCCGGCGCTCGCTGCTCCCGCGGCCCGCGCCATGCCCTCCTACACGGTCACCGTGGCCACTGGC
	AGCCAGTGGTTCGCCGGCACTGACGACTACATCTACCTCAGCCTCGTGGGCTCGGCGGGCTGCAGCGA
	GAAGCACCTGCTGGACAAGCCCTTCTACAACGACTTCGAGCGTGGCGCGGTGAGCGCGGGCGGGGCA
	CGGGTGGAGCGCGGGCTGAGGTGCGTCCGGGACCCGGTTTGGACGGCAGAGGCCTGGGCGGGGGCG
	CCGAGGGCCCGTCGGGGCGGCCCGGACAGGACTGGGGGTGTCCAGGACCCTGTCAGGGAGGGCAGA

Table S1 Continued

Expression vector construction	Primer sequence (5′→3′)
	ACTGCGGTGGGGCGTGCCCTGGGCTCCCAGTGGCCGGTGGGTACCCTGGTGGGCAAGCGTCCAGGACC
	CCTCGCGGCGGCCGCGACCCCTGTCGGAAACGGAGACTTCCCGCGTGCCGCCTGCAAGGCGTCTTCCCT
	GGGAGGAGAAGGCCCAAGGTTTCCCCTCCACTTCAAGATCTGGGCTCCGAGGCTCCGGAGCCCCCTTCA
	CTGGGCCGCGTTTCTTGTGCCACCTCTTCCTTAGGCTCCTTCTGCAGGTCGCACAGGGAGGGAGAGGCAG
	ATGAGTCATGCTCACAGAACTCTGAGGGCACCTCGGAGCAGCCAGCTGTGAGCCGTGGGGAAGGGTGCA
	CGTCAGCGGGTCCAGAGGCCTGAGTTCTGTCCAGACATGACCACCAGCTCACTCTACCGTCTGTCTGGGC
	CTCAGTTTCCCCAGCTGAAAATGGAGGGTTGGCTTCAGTCTGGTCC
Site-direct mutant vector construction	Primer sequence (5′→3′)
pGL4.10-rs702365[G]	Forward: CTGCTCTCCCTCCTGCCTCCCCCCTCCTT
	Reverse: AAGGAGGGGGGAGGCAGGAGGGAGAGCAG
EMSA	Probe sequence (5′→3′)
EMSA-rs702365[C]	Forward: Biotin-GCTCTCCCTCCTCCCTCCCCCCTCC
	Reverse: Biotin-GGAGGGGGGAGGGAGGAGGGAGAGC
EMSA-rs702365[G]	Forward: Biotin-GCTCTCCCTCCTGCCTCCCCCCTCC
	Reverse: Biotin-GGAGGGGGGAGGCAGGAGGGAGAGC
Quantitative real-time PCR	Primer sequence (5′→3′)
ALOX5	Forward: GGAGAACCTGTTCATCAACCGC
	Reverse: CAGGTCTTCCTGCCAGTGATTC
GAPDH	Forward: AACGGATTTGGTCGTATTGGG
	Reverse: TGATTTTGGAGGGATCTCGC
siRNA transduction	Sense sequence (5′→3′)
siControl	GCGACGAUCUGCCUAAGAUTT
siALOX5#1	GCAGGAAGACCUGAUGUUUTT
siALOX5#2	CUGAGCGCAACAAGAAGAATT

Table S2 Associations of 169 genetic variations and OS of rectal cancer patients based on the additive model

Gene	Chr.	Genetic variations	Allele	MAF in CHB	Crude		Adjusted[†]	
					HR (95% CI)	*P*-value	HR (95% CI)	*P*-value
APAF1	12	rs10860361	G > A	0.48	1.04 (0.76–1.43)	0.802	1.04 (0.76–1.41)	0.822
APAF1	12	rs11296996	A > del	0.37	1.03 (0.75–1.43)	0.848	1.06 (0.76–1.47)	0.725
APAF1	12	rs12581708	A > G	0.29	1.02 (0.71–1.45)	0.924	1.13 (0.78–1.63)	0.514
APAF1	12	rs2118851[‡]	C > G	0.21	0.98 (0.66–1.45)	0.918	1.15 (0.78–1.71)	0.481
APAF1	12	rs74619561	G > A	0.15	0.96 (0.58–1.59)	0.887	0.80 (0.48–1.34)	0.398
BAK	6	rs5745568	G > T	0.25	0.85 (0.59–1.22)	0.380	0.77 (0.53–1.12)	0.164
BAK	6	rs74499662	GTAA > del	0.41	0.90 (0.64–1.25)	0.520	0.96 (0.68–1.35)	0.798
BAX	19	rs4645904	G > A	0.49	1.22 (0.87–1.71)	0.246	1.21 (0.86–1.70)	0.266
BAX	19	rs905238	A > G	0.37	0.79 (0.56–1.13)	0.197	0.82 (0.57–1.18)	0.285
BCL2	18	rs1944423[‡]	A > G	0.41	0.82 (0.58–1.17)	0.279	0.82 (0.57–1.18)	0.283
BID	22	rs366542	T > C	0.47	1.05 (0.76–1.45)	0.787	1.05 (0.75–1.47)	0.757
BID	22	rs148719901[‡]	del > ins	0.50	1.12 (0.81–1.54)	0.491	1.08 (0.78–1.50)	0.632
BID	22	rs8190256[‡]	T > G	0.11	1.13 (0.71–1.81)	0.602	1.17 (0.73–1.89)	0.520
CASP3	**4**	**rs113420705[‡]**	**C > T**	**0.32**	**1.45 (1.05–2.00)**	**0.026**	**1.54 (1.09–2.16)**	**0.013**
CASP3	**4**	**rs4647700**	**GAG > del**	**0.12**	**1.60 (1.07–2.39)**	**0.022**	**1.52 (1.01–2.29)**	**0.045**
CASP3	4	rs1049216	G > A	0.21	0.93 (0.62–1.38)	0.704	0.96 (0.64–1.45)	0.859
CASP3	4	rs4647602	T > G	0.38	1.25 (0.92–1.70)	0.154	1.28 (0.93–1.77)	0.126
CASP6	4	rs1042891	G > A	0.47	1.12 (0.81–1.56)	0.477	1.30 (0.94–1.81)	0.116
CASP6	4	rs71595508	del > ins	0.18	1.04 (0.71–1.52)	0.841	0.96 (0.65–1.43)	0.854
CASP6	4	rs7682236[‡]	A > G	0.40	1.00 (0.72–1.40)	0.986	1.09 (0.77–1.53)	0.641
CASP6	4	rs7660005[‡]	T > C	0.49	0.88 (0.63–1.23)	0.455	0.84 (0.60–1.18)	0.308
CASP7	10	rs10787498	T > G	0.19	1.21 (0.85–1.73)	0.292	1.26 (0.86–1.84)	0.231
CASP7	10	rs1127687	G > A	0.20	0.87 (0.58–1.32)	0.518	0.95 (0.61–1.46)	0.804
CASP7	10	rs12415607	C > A	0.42	1.04 (0.75–1.44)	0.825	0.98 (0.70–1.38)	0.910
CASP7	10	rs7921977[‡]	T > C	0.19	0.74 (0.47–1.16)	0.186	0.70 (0.45–1.09)	0.118
CASP7	10	rs10553596	TT > del	0.21	1.13 (0.78–1.62)	0.519	1.27 (0.86–1.86)	0.225
CASP7	**10**	**rs12263370[‡]**	**G > A**	**0.14**	**1.75 (1.18–2.58)**	**0.005**	**1.81 (1.23–2.66)**	**0.003**
CASP7	10	rs2227310	C > G	0.43	1.03 (0.74–1.44)	0.869	0.98 (0.69–1.39)	0.918
CASP7	10	rs12247479	G > A	0.11	1.51 (0.97–2.35)	0.066	1.47 (0.93–2.33)	0.101
CASP8	2	rs13113[‡]	T > A	0.48	1.12 (0.80–1.57)	0.503	1.13 (0.80–1.59)	0.501
CASP8	2	rs3729647	G > C	0.19	1.06 (0.71–1.57)	0.783	1.09 (0.73–1.61)	0.673
CASP8	2	rs3769823	G > A	0.31	0.96 (0.68–1.36)	0.831	0.95 (0.67–1.36)	0.796
CASP9	1	rs1052576[‡]	C > T	0.38	1.11 (0.80–1.56)	0.526	1.14 (0.81–1.60)	0.446
CASP9	1	rs12741552[‡]	C > T	0.45	0.96 (0.70–1.33)	0.820	0.94 (0.68–1.31)	0.729

Table S2 Continued

Gene	Chr.	Genetic variations	Allele	MAF in CHB	Crude		Adjusted[†]	
					HR (95% CI)	*P*-value	HR (95% CI)	*P*-value
CASP10	2	rs13006529	T > A	0.18	0.98 (0.65–1.47)	0.919	1.02 (0.69–1.52)	0.909
FAS	10	rs1468063	C > T	0.39	1.22 (0.88–1.70)	0.233	1.14 (0.81–1.59)	0.453
FAS	10	rs4934431	C > T	0.39	1.11 (0.81–1.53)	0.504	1.04 (0.75–1.45)	0.805
FAS	10	rs1800682[‡]	A > G	0.38	0.97 (0.70–1.36)	0.873	0.97 (0.69–1.35)	0.835
FASL	1	rs763110	C > T	0.22	0.95 (0.66–1.36)	0.759	1.05 (0.71–1.55)	0.803
TNFR	12	rs4149570	C > A	0.47	1.02 (0.75–1.40)	0.883	0.97 (0.71–1.32)	0.834
TNFR	12	rs4149588[‡]	del > ins	0.27	0.77 (0.53–1.12)	0.172	0.84 (0.58–1.22)	0.356
TNFR	12	rs767455	T > C	0.12	1.34 (0.86–2.09)	0.195	1.53 (0.96–2.44)	0.073
TRAIL	3	rs112822654[‡]	GTGA > del	0.40	0.94 (0.68–1.30)	0.714	0.97 (0.70–1.35)	0.870
TRAIL	3	rs388515[‡]	A > G	0.35	1.00 (0.72–1.39)	0.982	0.99 (0.69–1.41)	0.954
TRAIL	3	rs12488654	A > G	0.50	1.13 (0.81–1.57)	0.467	1.20 (0.84–1.72)	0.323
TRAIL	3	rs1131532	G > A	0.43	0.92 (0.68–1.27)	0.627	0.94 (0.68–1.29)	0.687
TRAIL	3	rs9859259[‡]	C > A	0.50	0.89 (0.64–1.23)	0.475	0.84 (0.60–1.18)	0.316
TRAILR1	8	rs13255394	T > C	0.28	0.82 (0.57–1.19)	0.305	0.89 (0.61–1.30)	0.535
TRAILR2	8	rs1047266	G > A	0.26	1.00 (0.69–1.46)	0.984	1.00 (0.70–1.44)	0.988
TRAILR2	8	rs1047275	C > G	0.46	1.14 (0.84–1.55)	0.408	1.06 (0.78–1.44)	0.722
TRAILR2	8	rs2889	G > A	0.49	0.95 (0.69–1.31)	0.750	0.99 (0.72–1.37)	0.949
TRAILR2	**8**	**rs11784599**	**C > A**	**0.12**	**0.69 (0.43–1.11)**	**0.127**	**0.61 (0.37–0.98)**	**0.042**
AIM2	1	rs2298803	T > C	0.29	0.93 (0.65–1.34)	0.708	0.91 (0.62–1.33)	0.627
CASP1	11	rs1977989[‡]	A > G	0.27	1.12 (0.75–1.67)	0.567	1.27 (0.84–1.92)	0.256
CASP11	12	rs10880867[‡]	A > G	0.33	1.14 (0.81–1.60)	0.452	1.22 (0.85–1.74)	0.284
CASP11	12	rs148361648[‡]	TA > del	0.17	0.85 (0.55–1.31)	0.459	0.94 (0.60–1.45)	0.774
CASP11	12	rs10880868[‡]	C > T	0.12	1.11 (0.71–1.75)	0.643	1.12 (0.70–1.79)	0.645
CASP11	12	rs7315731	T > A	0.43	1.04 (0.74–1.45)	0.823	1.06 (0.75–1.48)	0.749
CASP11	12	rs11574952[‡]	C > T	0.45	0.97 (0.70–1.36)	0.880	0.97 (0.70–1.34)	0.853
CASP11	12	rs138471442[‡]	ins > del	0.33	0.98 (0.69–1.40)	0.919	0.97 (0.68–1.38)	0.862
CASP4	**11**	**rs571407**	**T > C**	**0.41**	**0.61 (0.43–0.86)**	**0.005**	**0.57 (0.40–0.81)**	**0.002**
CASP4	**11**	**rs612987**	**T > C**	**0.48**	**0.67 (0.48–0.94)**	**0.019**	**0.65 (0.46–0.93)**	**0.017**
CASP4	11	rs11226565	A > G	0.10	0.48 (0.25–0.93)	0.030	0.57 (0.29–1.11)	0.099
CASP4	**11**	**rs623114**	**A > G**	**0.41**	**0.70 (0.50–0.98)**	**0.039**	**0.64 (0.45–0.92)**	**0.014**
CASP4	**11**	**rs543923[‡]**	**C > T**	**0.19**	**0.66 (0.43–1.02)**	**0.063**	**0.61 (0.39–0.95)**	**0.030**
CASP4	11	rs579408[‡]	T > A	0.27	0.68 (0.44–1.05)	0.080	0.68 (0.44–1.05)	0.083
CASP5	11	rs540819	T > A	0.41	0.75 (0.54–1.06)	0.104	0.72 (0.51–1.02)	0.066
CASP5	11	rs507879	T > C	0.23	0.84 (0.53–1.33)	0.462	0.97 (0.61–1.56)	0.905

Table S2 Continued

Gene	Chr.	Genetic variations	Allele	MAF in CHB	Crude		Adjusted[†]	
					HR (95% CI)	*P*-value	HR (95% CI)	*P*-value
GSDMD	8	rs10099546[‡]	A > G	0.43	1.19 (0.85–1.66)	0.303	1.26 (0.89–1.78)	0.193
GSDMD	8	rs13266821[‡]	C > T	0.33	1.17 (0.82–1.65)	0.382	1.21 (0.85–1.72)	0.302
GSDMD	8	rs11782753	C > T	0.18	0.97 (0.63–1.49)	0.876	0.80 (0.51–1.26)	0.336
GSDME	**7**	**rs2954558**	**C > T**	**0.19**	**1.41 (0.99–2.03)**	**0.059**	**1.52 (1.06–2.19)**	**0.023**
GSDME	7	rs12540919	C > T	0.16	1.32 (0.88–1.98)	0.179	1.37 (0.90–2.07)	0.140
GSDME	7	rs4719777[‡]	C > G	0.33	0.81 (0.56–1.17)	0.255	0.79 (0.54–1.15)	0.213
GSDME	7	rs2074142[‡]	T > C	0.30	0.80 (0.54–1.18)	0.263	0.84 (0.55–1.27)	0.404
GSDME	7	rs2237314	T > C	0.10	1.27 (0.82–1.97)	0.284	1.27 (0.81–1.98)	0.291
GSDME	7	rs2237323[‡]	G > A	0.16	1.21 (0.81–1.82)	0.350	1.23 (0.83–1.82)	0.309
GSDME	7	rs17149888	G > A	0.19	0.83 (0.54–1.26)	0.373	0.77 (0.51–1.18)	0.238
GSDME	7	rs2257061	C > T	0.17	1.16 (0.80–1.70)	0.433	1.28 (0.86–1.91)	0.225
GSDME	7	rs12700545	G > C	0.50	0.88 (0.63–1.23)	0.455	0.80 (0.58–1.12)	0.199
GSDME	7	rs2237311	G > A	0.33	0.89 (0.63–1.27)	0.519	0.89 (0.62–1.27)	0.515
GSDME	7	rs1476521	A > G	0.32	1.10 (0.79–1.54)	0.563	1.23 (0.88–1.71)	0.228
GSDME	7	rs2521769	A > G	0.16	0.90 (0.54–1.48)	0.673	0.98 (0.59–1.64)	0.948
GSDME	7	rs2237317	C > T	0.23	0.94 (0.65–1.36)	0.757	0.90 (0.62–1.31)	0.574
GSDME	7	rs754554	G > T	0.50	1.00 (0.72–1.39)	0.978	0.98 (0.70–1.36)	0.897
GSDME	7	rs2521772[‡]	T > A	0.19	1.00 (0.65–1.52)	0.991	0.97 (0.63–1.49)	0.891
NLRP3	1	rs12239046[‡]	C > T	0.38	1.36 (0.99–1.88)	0.059	1.33 (0.95–1.85)	0.095
NLRP3	1	rs12143966[‡]	A > G	0.48	1.35 (0.97–1.88)	0.071	1.34 (0.95–1.89)	0.091
NLRP3	1	rs36021952[‡]	G > A	0.10	0.66 (0.39–1.13)	0.132	0.61 (0.35–1.07)	0.084
NLRP3	1	rs34691535[‡]	del > ins	0.42	1.14 (0.82–1.58)	0.448	1.15 (0.82–1.61)	0.421
NLRP3	1	rs10925017	G > A	0.33	1.12 (0.79–1.58)	0.521	1.04 (0.74–1.45)	0.835
NLRP3	1	rs10754557	A > G	0.35	0.93 (0.65–1.32)	0.684	0.91 (0.63–1.31)	0.605
NLRP3	1	rs12048215[‡]	A > G	0.30	0.96 (0.65–1.43)	0.859	1.06 (0.70–1.60)	0.788
NLRP3	1	rs3806268[‡]	A > G	0.49	1.01 (0.73–1.39)	0.945	1.09 (0.79–1.52)	0.586
NLRP3	1	rs7525979	C > T	0.16	0.99 (0.66–1.48)	0.947	1.09 (0.72–1.65)	0.693
ALOX15	17	rs11078528[‡]	G > A	0.34	0.84 (0.59–1.22)	0.362	0.84 (0.57–1.23)	0.370
ALOX15	17	rs916055	A > G	0.43	0.86 (0.61–1.21)	0.383	0.89 (0.62–1.29)	0.548
ALOX15	17	rs1965923[‡]	C > G	0.45	0.98 (0.69–1.39)	0.912	1.07 (0.74–1.55)	0.717
ALOX15	17	rs2619117	G > A	0.47	1.13 (0.82–1.57)	0.453	1.06 (0.75–1.50)	0.733
ALOX15	17	rs2619112	G > A	0.45	0.87 (0.62–1.23)	0.429	1.00 (0.70–1.43)	0.998
ALOX5	**10**	**rs702365[‡]**	**G > C**	**0.33**	**0.66 (0.47–0.93)**	**0.019**	**0.67 (0.47–0.96)**	**0.028**
ALOX5	**10**	**rs2242332**	**C > T**	**0.22**	**0.59 (0.39–0.89)**	**0.013**	**0.57 (0.38–0.87)**	**0.010**

Table S2 Continued

Gene	Chr.	Genetic variations	Allele	MAF in CHB	Crude		Adjusted†	
					HR (95% CI)	*P*-value	HR (95% CI)	*P*-value
ALOX5	**10**	**rs4948673‡**	**T > A**	**0.18**	**0.56 (0.35–0.90)**	**0.017**	**0.55 (0.34–0.89)**	**0.015**
ALOX5	10	rs10900213	T > G	0.41	0.86 (0.62–1.20)	0.376	0.86 (0.62–1.20)	0.367
ALOX5	10	rs78334475	C > G	0.15	0.90 (0.52–1.57)	0.721	0.79 (0.45–1.38)	0.404
ALOX5	10	rs12264801‡	G > A	0.40	0.80 (0.57–1.12)	0.186	0.90 (0.64–1.28)	0.568
ALOX5	10	rs3780905	C > T	0.49	1.08 (0.78–1.49)	0.642	1.10 (0.79–1.53)	0.581
ALOX5	10	rs11439622	C > CT	0.16	0.97 (0.63–1.51)	0.906	0.90 (0.57–1.42)	0.658
ALOX5	10	rs4949001‡	G > A	0.46	0.96 (0.69–1.32)	0.791	0.96 (0.69–1.33)	0.801
ALOX5	10	rs4948672‡	G > C	0.14	1.09 (0.71–1.68)	0.682	1.05 (0.68–1.61)	0.841
BECN1	17	rs11552192	A > T	0.16	1.00 (0.67–1.49)	0.998	1.00 (0.67–1.49)	0.997
DMT1	12	rs224446‡	C > T	0.45	0.85 (0.62–1.16)	0.300	0.86 (0.62–1.18)	0.339
DMT1	12	rs224575‡	T > C	0.42	0.96 (0.69–1.34)	0.820	0.89 (0.63–1.25)	0.496
DMT1	12	rs3809320‡	T > A	0.33	1.00 (0.70–1.43)	0.994	0.88 (0.62–1.27)	0.500
EIF2S1	14	rs33969193‡	C > CT	0.41	1.00 (0.73–1.37)	0.983	0.96 (0.68–1.34)	0.788
EIF2S1	14	rs8016024‡	T > C	0.43	1.04 (0.76–1.43)	0.789	0.97 (0.69–1.36)	0.857
FADS2	11	rs526126‡	C > G	0.11	0.83 (0.47–1.46)	0.515	0.76 (0.41–1.38)	0.366
FADS2	11	rs35622765	A > ACT	0.24	0.95 (0.63–1.44)	0.808	0.92 (0.60–1.40)	0.682
FADS2	11	rs74771917‡	C > T	0.16	0.91 (0.55–1.50)	0.705	0.93 (0.56–1.56)	0.793
FADS2	11	rs174602	T > C	0.26	1.00 (0.68–1.46)	0.986	0.98 (0.67–1.43)	0.909
FADS2	11	rs174561	T > C	0.34	0.98 (0.68–1.41)	0.919	1.02 (0.71–1.47)	0.921
FTH1	11	rs1800009	C > T	0.15	0.92 (0.56–1.51)	0.737	0.87 (0.52–1.46)	0.608
FTH1	11	rs17156609	G > A	0.17	1.08 (0.71–1.65)	0.712	1.03 (0.67–1.59)	0.896
GPX4	**19**	**rs36207883‡**	**G > A**	**0.26**	**0.73 (0.51–1.04)**	**0.081**	**0.64 (0.44–0.93)**	**0.019**
GPX4	19	rs146333943	TGGGGTG > T	0.23	0.78 (0.50–1.20)	0.260	0.75 (0.48–1.19)	0.228
GPX4	19	rs2075711‡	A > G	0.48	1.20 (0.86–1.67)	0.288	1.12 (0.80–1.58)	0.504
GPX4	19	rs2074452‡	C > T	0.36	1.13 (0.81–1.57)	0.479	1.08 (0.77–1.52)	0.656
GPX4	19	rs713041‡	C > T	0.45	0.91 (0.66–1.25)	0.556	0.98 (0.70–1.37)	0.901
HO-1	**22**	**rs17883419‡**	**C > T**	**0.17**	**1.78 (1.23–2.57)**	**0.002**	**1.79 (1.24–2.58)**	**0.002**
HO-1	**22**	**rs2071749**	**G > A**	**0.25**	**0.57 (0.37–0.88)**	**0.010**	**0.55 (0.36–0.86)**	**0.008**
HO-1	22	rs2071746‡	T > A	0.45	0.82 (0.59–1.14)	0.229	0.78 (0.56–1.09)	0.150
HO-1	22	rs9607267	T > C	0.47	1.13 (0.83–1.56)	0.438	1.16 (0.84–1.59)	0.381
NQO1	16	rs2917670	T > C	0.15	0.70 (0.43–1.15)	0.163	0.78 (0.47–1.27)	0.311
NQO1	16	rs1800566‡	G > A	0.50	0.99 (0.73–1.35)	0.974	1.06 (0.78–1.44)	0.718
NQO1	16	rs2917669	G > A	0.35	1.19 (0.86–1.65)	0.282	1.06 (0.76–1.48)	0.722
NRF2	**2**	**rs73976300**	**C > T**	**0.13**	**0.60 (0.32–1.15)**	**0.127**	**0.50 (0.25–0.99)**	**0.046**

Table S2 Continued

Gene	Chr.	Genetic variations	Allele	MAF in CHB	Crude		Adjusted†	
					HR (95% CI)	*P*-value	HR (95% CI)	*P*-value
NRF2	2	rs147426191	GCAAA > G	0.42	0.82 (0.58–1.18)	0.288	0.79 (0.55–1.13)	0.194
NRF2	2	rs77684420‡	T > C	0.13	0.92 (0.55–1.56)	0.760	0.81 (0.47–1.39)	0.439
NRF2	2	rs36030784‡	A > C	0.10	1.07 (0.65–1.76)	0.784	1.16 (0.70–1.93)	0.569
NRF2	2	rs6726395	G > A	0.36	1.00 (0.70–1.41)	0.987	1.06 (0.75–1.49)	0.760
NRF2	2	rs10497511	A > G	0.29	0.97 (0.66–1.42)	0.870	0.99 (0.68–1.45)	0.973
NRF2	2	rs1962142	G > A	0.20	0.95 (0.64–1.43)	0.824	1.01 (0.67–1.50)	0.976
NRF2	2	rs12471433‡	A > C	0.48	0.97 (0.70–1.34)	0.856	1.00 (0.72–1.39)	0.993
PTGS2	1	rs689466‡	T > C	0.47	1.22 (0.87–1.71)	0.257	1.22 (0.87–1.72)	0.256
SLC3A2	11	rs10897300‡	A > G	0.12	0.52 (0.27–0.99)	0.046	0.57 (0.30–1.09)	0.090
SLC3A2	11	rs34722625‡	T > TG	0.47	0.83 (0.60–1.16)	0.271	0.77 (0.54–1.09)	0.139
SLC3A2	11	rs201042920‡	T > G	0.50	0.85 (0.61–1.18)	0.319	0.78 (0.55–1.11)	0.170
SLC3A2	11	rs34436839‡	CAG > C	0.15	1.20 (0.76–1.91)	0.432	1.33 (0.82–2.16)	0.254
SLC3A2	11	rs489381‡	G > A	0.16	0.76 (0.44–1.29)	0.302	0.73 (0.42–1.28)	0.274
SLC3A2	11	rs12794763‡	T > G	0.32	1.08 (0.74–1.58)	0.676	1.13 (0.77–1.64)	0.539
SLC3A2	11	rs2070870‡	T > C	0.12	1.10 (0.67–1.81)	0.709	1.13 (0.67–1.89)	0.654
SLC3A2	11	rs79118350	CG > C	0.30	1.00 (0.70–1.43)	0.989	1.04 (0.71–1.51)	0.848
SLC3A2	11	rs2282477‡	T > C	0.23	0.95 (0.66–1.38)	0.805	1.04 (0.72–1.50)	0.855
SLC7A11	4	rs4330397	C > G	0.36	0.80 (0.57–1.13)	0.203	0.74 (0.52–1.04)	0.086
SLC7A11	4	rs4353954‡	T > A	0.24	1.33 (0.92–1.90)	0.125	1.22 (0.85–1.76)	0.282
SLC7A11	4	rs79652067‡	G > A	0.42	0.85 (0.62–1.17)	0.325	0.84 (0.61–1.16)	0.290
SLC7A11	4	rs62324387	T > C	0.26	1.09 (0.75–1.59)	0.659	1.21 (0.82–1.80)	0.336
SLC7A11	4	rs12507560	G > T	0.49	1.18 (0.84–1.65)	0.340	1.17 (0.83–1.65)	0.364
SLC7A11	4	rs142705636	T > TAA	0.10	1.09 (0.64–1.87)	0.753	0.88 (0.50–1.54)	0.656
SLC7A11	4	rs35372640‡	C > T	0.32	0.92 (0.64–1.31)	0.639	0.94 (0.65–1.36)	0.745
SLC7A11	4	rs35108180‡	G > A	0.40	1.12 (0.79–1.58)	0.532	0.95 (0.66–1.38)	0.796
SLC7A11	4	rs4429772‡	A > G	0.33	0.93 (0.66–1.33)	0.703	1.05 (0.73–1.51)	0.801
SLC7A11	4	rs4501269‡	G > A	0.36	1.03 (0.75–1.43)	0.836	1.04 (0.75–1.44)	0.830
SLC7A11	4	rs77293413	TA > T	0.44	0.96 (0.69–1.34)	0.818	0.97 (0.70–1.35)	0.860
SLC7A11	4	rs4863771	G > T	0.27	1.18 (0.83–1.69)	0.350	1.02 (0.71–1.48)	0.898
SLC7A11	4	rs7674870‡	A > G	0.32	1.07 (0.78–1.48)	0.665	0.98 (0.70–1.38)	0.916
SLC7A11	4	rs6832143‡	A > G	0.37	1.12 (0.81–1.55)	0.479	0.99 (0.70–1.40)	0.950
TFRC	3	rs406271‡	T > C	0.17	1.14 (0.74–1.75)	0.564	1.09 (0.68–1.72)	0.728
TFRC	3	rs3817672‡	C > T	0.14	1.04 (0.66–1.64)	0.881	1.05 (0.65–1.69)	0.840

MAF, minor allele frequency; CHB, Chinese Han Beijing population; HR, hazard ratio; CI, confidence interval. †Adjusted for gender, age, clinical stage, tumor grade, KPS, surgical procedure, and tumor location. ‡Some samples were not successfully genotyped. The bold parts mean that the genetic variations are significantly associated with OS in Cox regression models after adjusted.

Table S3 Relationships between four genetic variations in our previous study and OS of rectal cancer patients in co-dominant and dominant models

Genotype	Alive (%)	Death (%)	Crude		Adjusted†	
			HR (95% CI)	*P*-value	HR (95% CI)	*P*-value
**rs202195689**						
CCCCA-CCCCA	146 (64.6)	37 (50.0)	1.00 (reference)	-	1.00 (reference)	-
CCCCA-del	72 (31.9)	33 (44.6)	1.74 (1.09–2.78)	0.021	1.85 (1.13–3.04)	0.014
del-del	8 (3.5)	4 (5.4)	1.73 (0.62–4.85)	0.298	1.34 (0.47–3.83)	0.579
CCCCA-del + del-del	80 (35.4)	37 (50.0)	1.74 (1.10–2.74)	0.017	1.77 (1.10–2.84)	0.018
**rs5743030‡**						
GG	165 (73.7)	67 (91.8)	1.00 (reference)	-	1.00 (reference)	-
GA	54 (24.1)	6 (8.2)	0.32 (0.14–0.73)	0.007	0.29 (0.12–0.67)	0.004
AA	5 (2.2)	0 (0.0)	-	0.959	-	0.961
GA + AA	59 (26.3)	6 (8.2)	0.29 (0.13–0.67)	0.004	0.26 (0.11–0.62)	0.002
**rs4920657**						
TT	126 (55.8)	53 (71.6)	1.00 (reference)	-	1.00 (reference)	-
TA	81 (35.8)	18 (24.3)	0.57 (0.33–0.97)	0.039	0.51 (0.29–0.88)	0.016
AA	19 (8.4)	3 (4.1)	0.44 (0.14–1.41)	0.166	0.38 (0.12–1.23)	0.105
TA + AA	100 (44.2)	21 (28.4)	0.55 (0.33–0.91)	0.019	0.48 (0.29–0.81)	0.006
**rs5743100‡**						
GG	158 (71.5)	66 (89.2)	1.00 (reference)	-	1.00 (reference)	-
GT	59 (26.7)	8 (10.8)	0.37 (0.18–0.78)	0.008	0.35 (0.16–0.73)	0.005
TT	4 (1.8)	0 (0.0)	-	0.962	-	0.965
GT + TT	63 (28.5)	8 (10.8)	0.35 (0.17–0.73)	0.005	0.32 (0.15–0.67)	0.003

HR, hazard ratio; CI, confidence interval. †Adjusted for gender, age, clinical stage, tumor grade, KPS, surgical procedure, and tumor location. ‡Some samples were not successfully genotyped.

Table S4 Associations between different number of risk genotypes and OS in patients with rectal cancer receiving postoperative CRT for joint analysis of *CASP4* rs571407, *ALOX5* rs2242332 and *HO-1* rs17883419, hsa-miR-4274 rs202195689, and *PMS1* rs5743030

Number of risk genotypes‡	Alive (%)	Death (%)	Crude		Adjusted†	
			HR (95% CI)	*P*-value	HR (95% CI)	*P*-value
≤ 1	68 (30.5)	7 (9.7)	1.00 (reference)	-	1.00 (reference)	-
2	80 (35.9)	21 (29.2)	2.21 (0.94–5.21)	0.069	2.58 (1.08–6.13)	0.033
3	54 (24.2)	20 (27.8)	3.17 (1.34–7.49)	0.009	3.88 (1.63–9.22)	0.002
4	20 (9.0)	19 (26.4)	7.27 (3.05–17.3)	< 0.001	9.07 (3.66–22.47)	< 0.001
5	1 (0.4)	5 (6.9)	16.20 (5.12–51.25)	< 0.001	11.91 (3.6–39.35)	< 0.001
P-value for trend				< 0.001		< 0.001
≥ 4 of risk genotypes	21 (9.4)	24 (33.3)	8.20 (3.53–19.05)	< 0.001	9.68 (4.08–22.97)	< 0.001

HR, hazard ratio; CI, confidence interval. †Adjusted for gender, age, clinical stage, tumor grade, KPS, surgical procedure, and tumor location. ‡Risk genotypes were *CASP4* rs571407 TT, *ALOX5* rs2242332 CC and *HO-1* rs17883419 CT + TT, hsa-miR-4274 rs202195689 CCCCA-del + del-del and *PMS1* rs5743030 GG.
